# A genome-wide association study in multiple system atrophy

**DOI:** 10.1212/WNL.0000000000003221

**Published:** 2016-10-11

**Authors:** Anna Sailer, Sonja W. Scholz, Michael A. Nalls, Claudia Schulte, Monica Federoff, T. Ryan Price, Andrew Lees, Owen A. Ross, Dennis W. Dickson, Kin Mok, Niccolo E. Mencacci, Lucia Schottlaender, Viorica Chelban, Helen Ling, Sean S. O'Sullivan, Nicholas W. Wood, Bryan J. Traynor, Luigi Ferrucci, Howard J. Federoff, Timothy R. Mhyre, Huw R. Morris, Günther Deuschl, Niall Quinn, Hakan Widner, Alberto Albanese, Jon Infante, Kailash P. Bhatia, Werner Poewe, Wolfgang Oertel, Günter U. Höglinger, Ullrich Wüllner, Stefano Goldwurm, Maria Teresa Pellecchia, Joaquim Ferreira, Eduardo Tolosa, Bastiaan R. Bloem, Olivier Rascol, Wassilios G. Meissner, John A. Hardy, Tamas Revesz, Janice L. Holton, Thomas Gasser, Gregor K. Wenning, Andrew B. Singleton, Henry Houlden

**Affiliations:** Authors' affiliations are listed at the end of the article.

## Abstract

**Objective:**

To identify genetic variants that play a role in the pathogenesis of multiple system atrophy (MSA), we undertook a genome-wide association study (GWAS).

**Methods:**

We performed a GWAS with >5 million genotyped and imputed single nucleotide polymorphisms (SNPs) in 918 patients with MSA of European ancestry and 3,864 controls. MSA cases were collected from North American and European centers, one third of which were neuropathologically confirmed.

**Results:**

We found no significant loci after stringent multiple testing correction. A number of regions emerged as potentially interesting for follow-up at *p* < 1 × 10^−6^, including SNPs in the genes *FBXO47*, *ELOVL7*, *EDN1*, and *MAPT*. Contrary to previous reports, we found no association of the genes *SNCA* and *COQ2* with MSA.

**Conclusions:**

We present a GWAS in MSA. We have identified several potentially interesting gene loci, including the *MAPT* locus, whose significance will have to be evaluated in a larger sample set. Common genetic variation in *SNCA* and *COQ2* does not seem to be associated with MSA. In the future, additional samples of well-characterized patients with MSA will need to be collected to perform a larger MSA GWAS, but this initial study forms the basis for these next steps.

Multiple system atrophy (MSA) is an adult-onset neurodegenerative disorder of unknown cause. Clinical features include parkinsonism, cerebellar ataxia, pyramidal signs, and dysautonomia. MSA typically presents as a sporadic disease, although rare reports of familial occurrences have been described,^1–3^ and there is a greater risk of parkinsonian disorders in relatives of patients with MSA.^4^

The pathogenesis of MSA is largely unknown. Identification of α-synuclein-positive glial cytoplasmic inclusions as a neuropathologic hallmark provided the first clue that abnormal protein accumulation is involved in the development of MSA.^5^ Furthermore, it suggested a link to other neurodegenerative diseases that are characterized by α-synuclein deposition, commonly referred to as synucleinopathies. These include Parkinson disease (PD), MSA, dementia with Lewy bodies, pure autonomic failure, and neurodegeneration with brain iron accumulation type I.^6^

Additional evidence in support of shared pathogenic mechanisms among synucleinopathies was suggested by recent genetic findings demonstrating that variants at the *SNCA* locus, coding for the deposited α-synuclein protein, are associated with increased risk for PD and MSA.^7,8^ In addition, the (*MAPT*) H1 haplotype has been associated with MSA.^7,9^ More recently, the *COQ2* gene was reported to harbor mutations in familial MSA cases from Japan.^10^ However, follow-up studies in non-Asian ethnic cohorts were unable to replicate this finding.^11,12^ Thus, many prior observations require further evaluation.

To investigate whether common genetic risk factors play a role in MSA, we performed a genome-wide association study (GWAS) including 918 patients with MSA and 3,864 controls.

## METHODS

### Study design.

We performed a multicenter GWAS with patients with MSA of European ancestry and regionally matched controls. Collection sites for this study included brain banks and clinical centers in Germany, Austria, Netherlands, Denmark, the United Kingdom, Italy, Portugal, Spain, and the United States (table e-1 at Neurology.org). Since patients were recruited in different geographic regions across Europe and the United States, we matched cases with regional controls. Genotype data of healthy individuals from previous GWAS were obtained from Germany, the United Kingdom, the United States, and Italy. The MSA cases were grouped into 4 geographic regions and each case was matched with 4 controls from the same geographic region: UK and Irish MSA cases were matched with UK controls; Northern European MSA cases from Germany, Netherlands, Belgium, Austria, Denmark, and Sweden were matched with German controls; Southern European cases from Italy and Spain were matched with Italian controls; American MSA cases were matched with American controls. Matching was carried out using multidimensional scaling based on the first 2-component vectors for each subpopulation, derived from common variants assayed in both cases and controls (minor allele frequency [MAF] > 0.05). A flowchart of studied populations and quality control steps is shown in figure e-1.

### Study participants.

DNA samples from a total of 1,030 patients with MSA were collected for this study (details for each population are shown in table 1, demographics are listed in table e-2). Patients were clinically diagnosed with possible or probable MSA (n = 699) by movement disorders specialists, or pathologically with definite MSA (n = 331) by neuropathologists according to Gilman criteria.^13^ For controls, we used genotype data from 3,864 previously published neurologically normal individuals from the United Kingdom (n = 936 samples), Germany (n = 944 samples), United States (n = 794 samples), and Italy (n = 1,190 samples) (table 1). All controls were genotyped on Illumina BeadChips (Illumina, San Diego, CA, USA). Details about sample collection and genotyping procedures in control cohorts have been described elsewhere.^7,14–16^

**Table 1:**
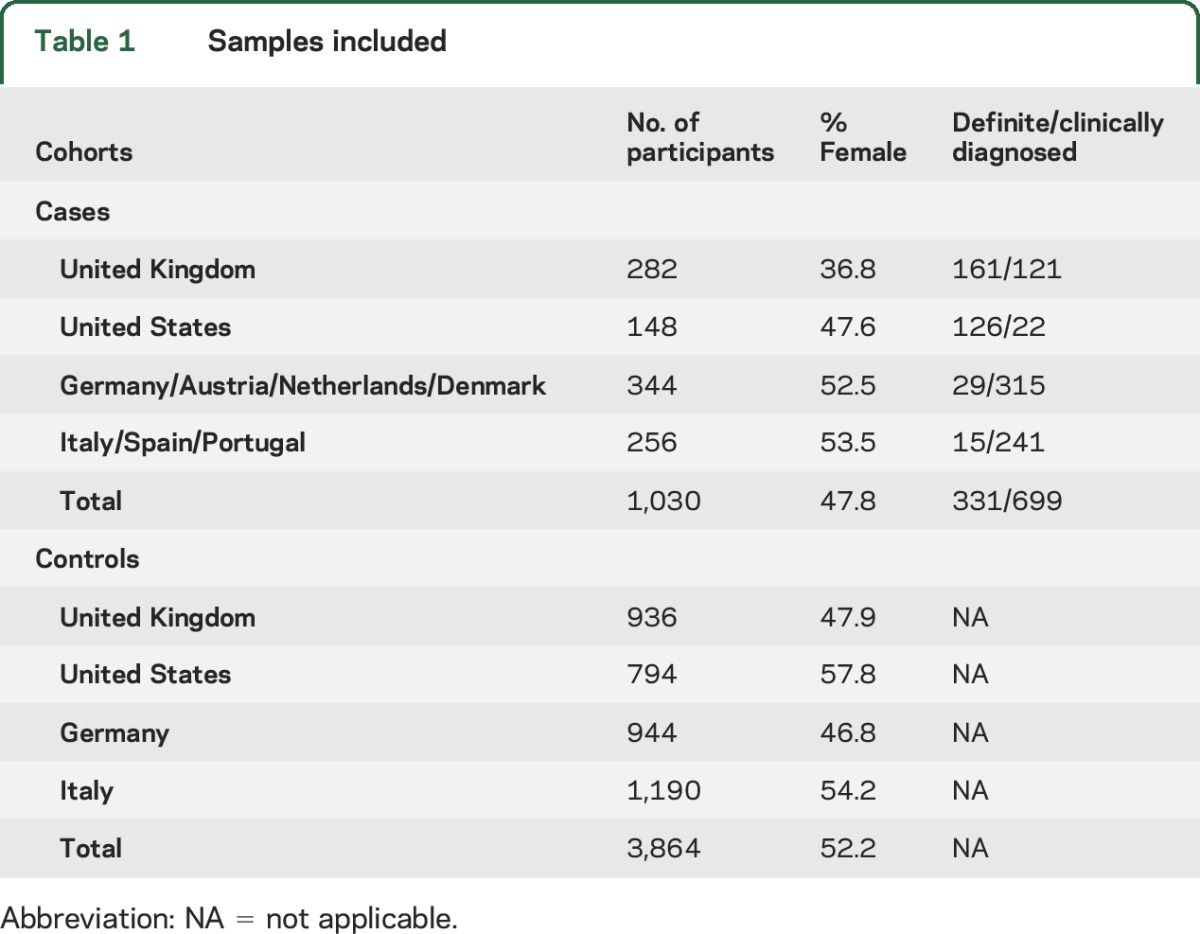
Samples included

### Standard protocol approvals, registrations, and patient consents.

The appropriate institutional review boards approved the study, and written informed consent was obtained for each participant.

### Genotyping.

Samples from 1,030 MSA cases were genotyped on 1 of 3 Illumina genotyping BeadChips (Human610-Quad v1, Human660W-Quad v1, or HumanOmniExpress-12 v1). Control samples were genotyped on the following BeadChips: UK WTCCC on Illumina Human1.2M-DuoCustom BeadChip; US, German, and Italian controls on Illumina BeadChips 550K version 1 chips or version 3.^7,14,16^ These chip versions have 343,783 unique single nucleotide polymorphisms (SNPs) in common, and only shared SNPs were used for downstream analyses.

### Statistical analysis.

#### Genotype calling.

For each of the 3 genotyping platforms, raw data were imported to GenomeStudio (v2010.1, genotyping module v1.6.3, Illumina) and genotype calls were reclustered using a no-call threshold of <0.15.

#### Quality control.

For quality control, we excluded samples with a genotyping call rate <95%, duplicate samples, cryptically related individuals (pi-hat threshold >0.2), individuals in whom the reported sex did not match the genotypic sex, and individuals with non-European ancestry as determined by multiple dimensional scaling analysis (samples deviating >6 SDs from the CEU/TSI population were excluded; see figure e-2). Next, we excluded SNPs with inaccurate cluster separation (cluster separation score <0.3), SNPs that were not shared among all 3 genotyping chip versions, and SNPs with an individual SNP call rate <95%. Following this step, we excluded SNPs with missingness by haplotype or phenotype that exceeded a significance of *p* value < 0.0001 or a significant deviation from Hardy-Weinberg equilibrium with a *p* value < 0.00001. Of the remaining SNPs, only those with a MAF >0.01 were used for downstream analyses.

#### Power calculations and heritability analysis.

Power calculations were performed for a GWAS testing 918 cases and 3,864 controls under a log-additive and a recessive model (QUANTO software v1.2.3). A minor allele frequency >1% and a 2-sided α = 5 × 10^−8^ were assumed. Under the log-additive model (figure e-3A), power analysis for this study indicated greater than 80% power to detect associated loci with an odds ratio greater than 1.8 at risk allele frequencies between 7% and 87%. We recently also performed a heritability analysis based on our GWAS data demonstrating that the heritability for MSA due to common coding variants is estimated to be between 2.1% and 6.7%.^17^

#### Genotype imputation.

About 4,903,804 SNPs were imputed based on haplotype reference data from the 1000 Genomes project (1000genomes.org/; December 2010 version). These data were derived from studying the genomic sequence of 104 participants of European ancestry. Imputations were performed for each of the 4 cohorts separately using MACH software as described elsewhere (version 1.0.16).^18,19^ SNPs with an *R*^2^ <0.3 and MAF <0.01 were excluded from further analysis as imputed genotypes below this threshold are likely to be of poor quality.

#### Association tests.

Due to the relatively diverse ancestries of European and US cohorts included in this study, we used principal component vectors 1 to 10 from a multidimensional scaling analysis generated in PLINK as covariates to adjust for possible population substructure in the logistic regression model used for the GWAS (figure e-4). The genomic inflation factor was λ = 1.057. For each SNP, *p* values and odds ratios under the additive models were calculated using Mach2dat software.^18,19^ Only SNPs exceeding the conservative Bonferroni threshold for multiple testing (*p* < 5 × 10^−8^) were considered genome-wide significant. We also performed a subanalysis for association in pathologically confirmed MSA cases (n = 295 cases after quality control) vs 3,864 controls. The genomic inflation factor in this subgroup was λ = 1.024.

#### SNCA SNP genotyping using a restriction enzyme assay.

SNP rs11931074, located downstream of the *SNCA* gene, was regenotyped using a restriction enzyme assay. Primers were designed to amplify the SNP and surrounding region. Restriction enzyme Bsr1 (New England Biolabs, Ipswich, MA) was used to cut the PCR product. The major allele was cut, whereas the minor allele remained uncut. Disease association was tested with a χ^2^ test.

#### Analysis of brain tissue quantitative trait loci.

For SNPs of interest, we attempted to infer functional consequences in frontal cortex and cerebellar tissue samples from neurologically normal individuals that were assayed for both genome-wide methylation and expression levels.^20^ These analyses may shed light on potential disease mechanisms for follow-up in future studies. We tested *cis* associations (any methylation or expression probes ±1 Mb from each SNP) in each of the datasets.^21^

## RESULTS

### Genotyping, imputation, and quality control.

After quality control procedures, the total study consisted of 918 cases and 3,864 controls. Of the MSA cases, 291 had a pathologically confirmed diagnosis. Samples were successfully genotyped for 267,998 SNPs and imputed to 4,903,804 SNPs. After removal of extreme ancestry outliers and using multidimensional scaling component vectors as covariates in regression models, only mild population stratification was evident based on genomic inflation factor calculations (λ = 1.057) (figure e-2).

### Association results.

**Figure 1:**
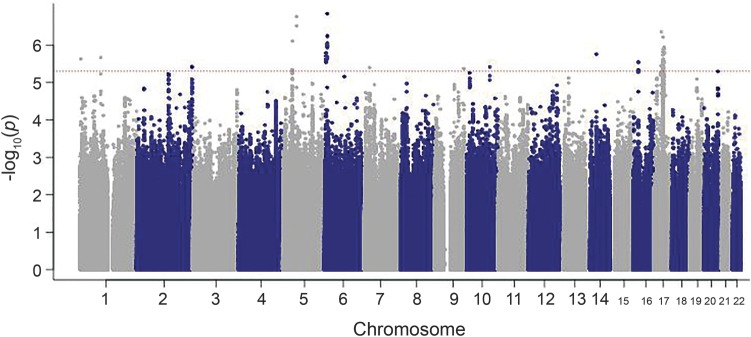
Manhattan plot showing association results in multiple system atrophy *p* Values under the additive association model are log transformed (y-axis) and plotted against the chromosomal position (x-axis). The dotted line indicates the threshold of potentially interesting single nucleotide polymorphisms.

Statistical analysis of association under an additive model was performed in the full case-control dataset (figure 1) and in the subset of MSA cases with neuropathologically confirmed diagnosis. Loci with the lowest *p* values (<1 × 10^−6^) in the full dataset and pathology confirmed cases are shown in tables 2 and e-3, respectively. In the full dataset, we identified 4 loci for future follow-up in a larger sample series at a *p* value <1 × 10^−7^ including the genes *FBXO47*, *ELOVL7*, *EDN1*, and *MAPT*. None of the tested SNPs surpassed the Bonferroni threshold for multiple testing (*p* value < 5 × 10^−8^) in the full dataset or in the subanalysis of pathologically confirmed cases.

**Table 2:**
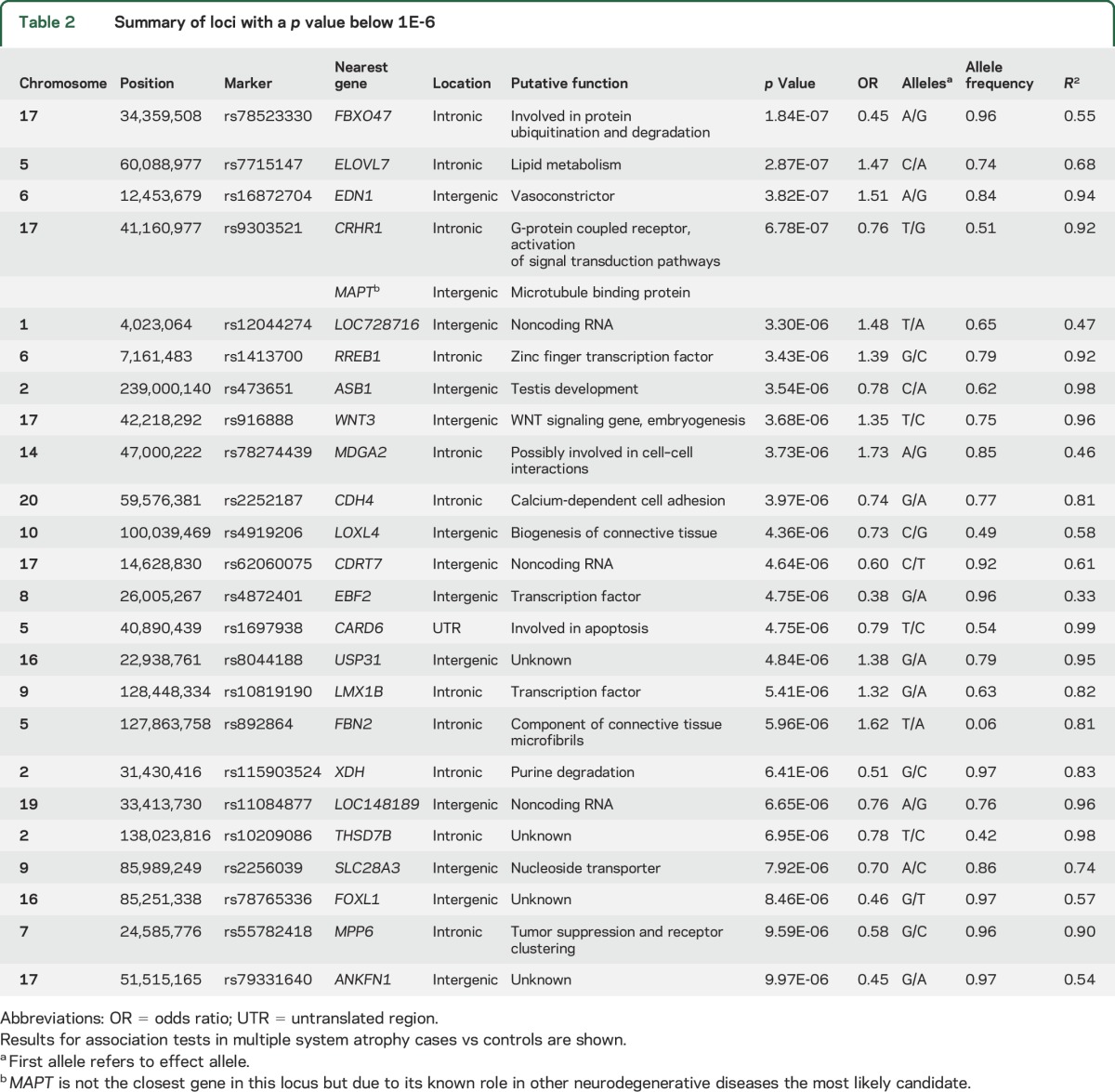
Summary of loci with a *p* value below 1E-6

### *COQ2* in the MSA GWAS data.

Recently, homozygous (or compound heterozygous) mutations in *COQ2* were reported in familial cases of MSA in Japan.^10^ While coding variants were also found at a higher frequency in sporadic MSA compared to controls, this finding did not replicate outside of Japanese MSA samples. We investigated the *COQ2* locus for common variation in our GWAS data. A total of 453 SNPs in the *COQ2* gene and the flanking region ±100 kb (build 36.3 positions) were tested for association. The most associated SNP was chr4:84473327 with a *p* value of 0.02169, which is far from genome-wide or even regional Bonferroni-adjusted significance. We therefore lack evidence that common genetic variation in the *COQ2* locus plays a major role in MSA risk in the European/Northern American population.

### SNCA rs11931074 SNP genotyping.

SNP rs11931074 in the *SNCA* locus (or rs3822086, which is in close linkage disequilibrium) has been linked to MSA in several smaller studies.^8,22,23^ This SNP is not represented on the OmniExpress Bead arrays; hence, it was not genotyped in any samples run on this array (n = 703) and consequently was not in the dataset of genotyped SNPs in common. Instead, the allele frequency was calculated postimputation, demonstrating that it was neither significantly associated in the entire dataset (*p* value = 0.4722) nor in the subgroup of definite MSA cases (*p* value = 0.4407). We decided to regenotype SNP rs11931074 in the MSA GWAS cohort using a restriction enzyme to exclude technical or imputation uncertainty (despite a high imputation score of 0.9670). Within the GWAS cohort, 906 MSA samples were available for regenotyping (table 3). In controls, data for this SNP were available from the bead array data. The minor allele of SNP rs11931074 was slightly more common in MSA cases vs controls (8.3% vs 7.3%) but again no significant association was found with MSA (χ^2^ = 2.6; *p* value = 0.27). This is in concordance with the imputed GWAS finding, but contradicts previous reports in the literature.^8,22,23^

**Table 3:**
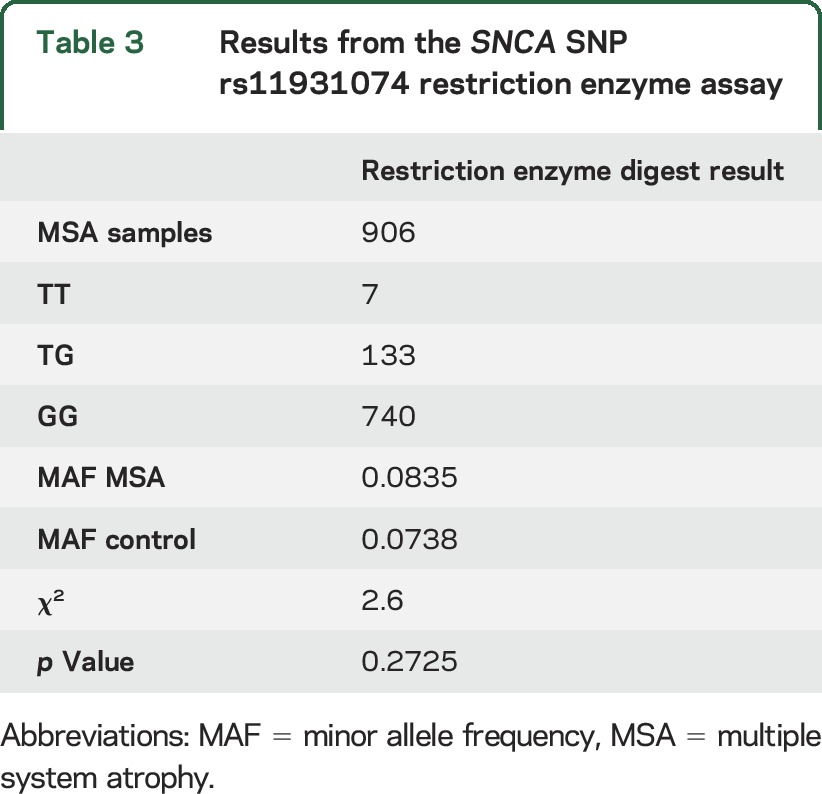
Results from the *SNCA* SNP rs11931074 restriction enzyme assay

### Analysis of brain tissue quantitative trait loci in candidate loci.

Twenty-one out of 24 SNPs of interest (*p* value < 1 × 10^−5^) passed quality control in the mRNA expression datasets, and 23 SNPs of interest passed quality control in the CpG methylation datasets. We tested multiple probes per SNP in each set of analyses. A total of 168 unique SNP–probe pairs were tested in the frontal cortex mRNA expression dataset, 165 pairs in the cerebellar mRNA expression dataset, and 391 pairs in the frontal cortex and cerebellar CpG methylation dataset. Associations were tested using linear regression adjusting for appropriate covariates and resulting *p* values were adjusted based on the false-discovery rate correction as previously described.^21^

After correcting for multiple testing, we found 8 significant associations between SNPs of interest and either CpG methylation or mRNA expression (table e-4). All of these were located on chromosome 17 and associated with 3 SNPs (rs78523330, rs9303521, and rs916888) in a region spanning 8 MB. SNP rs9393521 is located only 174 Kb upstream of MAPT, a gene previously suggested as a risk gene for MSA, and it is within a large 900 kb inversion polymorphism surrounding the MAPT locus.

## DISCUSSION

The contribution of common genetic factors to disease development has been established for many neurodegenerative conditions, which are complex, often sporadic conditions such as PD and progressive supranuclear palsy. In MSA, the identity of these contributing genetic factors remains almost entirely unknown. Here, we describe a GWAS investigating common genetic markers in 918 MSA cases and 3,864 controls for disease association.

Although none of the tested SNPs surpassed the stringent Bonferroni threshold, we identified 4 MSA regions with *p* values <1 × 10^−6^: *FBXO47*, *ELOVL7*, *EDN1*, and *MAPT*. These loci are most promising for further follow-up.

Among the most highly associated regions is the *MAPT* locus. *MAPT* is an intriguing candidate as it has already been implicated in a number of neurodegenerative diseases, particularly in tauopathies such as progressive supranuclear palsy, frontotemporal degeneration, and Alzheimer dementia.^24,25^ Although tau is not considered a key protein in MSA neuropathology, PD—which is also an α-synucleinopathy—has been found to be consistently associated with common variation in the *MAPT* locus.^7^ Furthermore, MSA was also previously found to be associated with the *MAPT* H1 haplotype in a small cohort of American MSA cases.^9^ These results, however, will need to be verified in a larger MSA cohort.

Located on chromosome 6, *EDN1* is part of the endothelin gene family, which functions in the maintenance of vascular tone. Studies have demonstrated the presence of endothelin peptides in nonvascular structures including epithelial cells, glia, and neurons.^26,27^ Given the phenotype of autonomic dysfunction or failure in patients with MSA, one may hypothesize that such pathophysiology may be associated with variation in *EDN1*.

*ELOVL7*, originating on chromosome 5, is involved in transferase activity. Chiefly, transferase is a condensing enzyme that acts as a catalyst for the synthesis of saturated and polyunsaturated very long chain fatty acids.^28,29^

*FBXO47* has a known association with papillary renal cell carcinoma.^30^ Located on chromosome 17, *FBXO47* promotes protein ubiquitination and degradation via phosphorylation, which are critical mechanisms in many neurodegenerative diseases.^30,31^ This locus could provide an important mechanism to MSA pathology in light of the oligodendroglial α-synuclein burden, but requires further study to draw any conclusions.

Common variation in the *COQ2* gene (recently found to be mutated in familial and sporadic probable MSA cases in Japan) did not show a significant association in the MSA GWAS data. This suggests that common genetic variation in this gene does not play a major role in disease pathogenesis in patients with MSA of European descent.

We were unable to replicate the previously reported association of variants at the *SNCA*.^8,22,23^ A Korean study was also unable to reproduce the association indicating possible differences between populations or an actual null result.^32^ The previously associated SNP rs11931074 has considerable variation in frequency among different populations. The reported risk allele has a frequency of about 7%–8% in our different European controls and in the European HapMap data.^33^ In contrast, the same allele is much more abundant in Asian and African populations (e.g., 58% in Japanese and 68% in Yorubans). Our study attempts to account for both interpopulation and intrapopulation heterogeneity through principal component analysis, and interpopulation heterogeneity of *SNCA* is a plausible explanation for the previous findings.

The quantitative trait analysis data suggest a complex risk locus on chromosome 17. This includes several SNPs in the region associated with multiple expression or methylation effects. Several of these genes are involved in pathways potentially relevant to MSA pathology: namely, the ribosylation protein (*ARL17A*), the ribosome protein (*RPL19*), and the proteasome protein (*PSMB3*).

The disease pathology of several neurodegenerative disorders illustrates a common theme of disrupted proteasomal protein clearance and degradation. Notably, *PSMB3* may be involved in trinucleotide repeat expansion—a phenomenon seen in several hereditary neurologic disorders (in particular autosomal dominant ataxias)—and it may be driving the trinucleotide expansion process.^34^ Supporting this hypothesis, there is considerable clinical overlap between MSA and several hereditary ataxias.^35,36^ Similarly, ribosomal dysfunction with aberrant protein synthesis may promote neurodegeneration.^37^ Interestingly, no alteration in expression of *MAPT*—the most obvious candidate gene in the chromosome 17 locus—was found. All but one associated QTL in MSA were found in cerebellar tissue (table e-4). Since the cerebellum is among the most disturbed brain regions in MSA, differences in expression in this region are consistent with an etiologic role in MSA.

There are a number of limitations to this study. First, although this represents by far the largest collection of MSA samples to date, the number of MSA cases is still relatively small for a GWAS. GWAS are designed to identify disease variants of relatively high frequency (MAF >1%) in a population operating under the common disease/common variant hypothesis.^38^ In PD, most identified risk factors have an odds ratio (OR) between 0.8 and 1.5.^39^ Similarly, in Alzheimer disease—apart from *APOE* (OR ∼4)—common genetic risk factors have low ORs ranging from 0.8 to 1.2. In these diseases, successful identification of significant loci was only feasible with considerably larger sample sizes than our study. However, with our current number we can reasonably exclude the existence of common risk alleles of large effect. A second limitation is the use of population control cohorts rather than age-matched controls, which increases the chance for population heterogeneity. We applied stringent corrections, including principal components from multidimensional scaling as covariates in our statistical model to address this concern. Third, the misdiagnosis rate of clinically ascertained patients, in particular those in early disease stages, can be as high as 38%.^40^ We addressed this concern by including a large number of pathology-proven cases (∼ one-third of all patients in this study). The inclusion of pathology-confirmed cases is particularly important for future follow-up studies in MSA.

Finally, a major challenge is that currently no additional large cohorts of MSA cases with a similar genetic background are available. A comparison of European and non-European cohorts may be informative with regard to interpopulation heterogeneity.

None of the studied variants were statistically significant after appropriate correction for multiple testing. However, we identified 4 promising loci that could reveal important insight into the disease pathways of MSA. Increasing the sample size of our GWAS will be key to the successful confirmation of these candidate loci and the identification of other risk genes.

## Supplementary Material

Data Supplement

Coinvestigators

Accompanying Editorial

## AUTHOR AFFILIATIONS

From the Departments of Molecular Neuroscience and Movement Disorders and The Reta Lila Weston Institute (A.S., A.L., K.M., N.E.M., L.S., V.C., H.L., S.S.O., N.W.W., J.A.H., T.R., J.L.H., H.H.), Department of Clinical Neuroscience (H.R.M.), and Sobell Department of Motor Neuroscience and Movement Disorders (N.Q., K.P.B.), UCL Institute of Neurology, London, UK; Klinik für Neurologie (A.S.), Universitätsklinik der Johann-Wolfgang-Goethe Universität Frankfurt, Germany; Laboratory of Neurogenetics, National Institute on Aging (S.W.S., M.A.N., M.F., T.R.P., B.J.T., A.B.S.), and Neurodegenerative Diseases Research Unit, National Institute of Neurological Disorders and Stroke (S.W.S.), National Institutes of Health, Bethesda, MD; Department of Neuroscience (S.W.S.), Georgetown University, Washington, DC; Department of Neurology (S.W.S.), Johns Hopkins Hospital, Baltimore, MD; Department of Neurodegenerative Diseases (C.S., T.G.), Hertie Institute for Clinical Brain Research, and German Center for Neurodegenerative diseases (DZNE), Tübingen, Germany; Department of Neuroscience (O.A.R., D.W.D.), Mayo Clinic, Jacksonville, FL; Division of Life Science (K.M.), Hong Kong University of Science and Technology, Hong Kong SAR, China; National Institute on Aging (L.F.), National Institutes of Health, Baltimore, MD; Department of Neuroscience (H.J.F., T.R.M.), Georgetown University, Washington, DC; Department of Neurology (G.D.), UKSH, Kiel Campus, Christian-Albrechts University Kiel, Germany; Division of Neurology (H.W.), Department of Clinical Sciences, University Hospital, Lund, Sweden; Istituto Clinico Humanitas (A.A.), Università Cattolica del Sacro Cuore, Milan, Italy; Service of Neurology (J.I.), University Hospital Marqués de Valdecilla (IDIVAL), University of Cantabria (UC), Santander, Spain; Institute of Neurology (W.P., G.K.W.), Medical University Innsbruck, Austria; Centre of Nervous Diseases (W.O.), Philipps-University of Marburg; Department of Neurology (G.U.H.), Technische Universität München; German Center for Neurodegenerative Diseases (DZNE) (G.U.H.), Munich; Department of Neurology (U.W.) and German Center for Neurodegenerative Diseases (U.W.), University Hospital of Bonn Medical Center, Germany; Parkinson Institute (S.G.), Istituti Clinici di Perfezionamento, Milan; Center for Neurodegenerative Diseases (M.T.P.), Department of Medicine and Surgery, Neuroscience Section, University of Salerno, Italy; Clinical Pharmacological Unit (J.F.), Instituto de Medicina Molecular, Faculty of Medicine, University of Lisbon, Portugal; Hospital Clinic (E.T.), University of Barcelona, Spain; Department of Neurology (B.R.B.), Donders Institute for Brain, Cognition and Behaviour, Radboud University Medical Centre, Nijmegen, Netherlands; Clinical Investigation Center CIC1436 (O.R.), Departments of Clinical Pharmacology and Neurosciences, INSERM and University Hospital of Toulouse, Faculty of Medicine; Centre de Référence Atrophie Multisystématisée (W.G.M.), CHU de Bordeaux, Pessac; and Université de Bordeaux (W.G.M.), Institut des Maladies Neurodégénératives, UMR 5293, Bordeaux, France.

## GLOSSARY

GWAS: genome-wide association study
MAF: minor allele frequency
MSA: multiple system atrophy
OR: odds ratio
PD: Parkinson disease
SNP: single nucleotide polymorphism
