# Supplementary material for: A genome-wide association study in multiple system atrophy
Source: Neurology. 2016 Oct 11;87(15):1591–8. doi: 10.1212/WNL.0000000000003221 (PMC5067544; doi:10.1212/WNL.0000000000003221)
Supplement: Data Supplement [file supp_87_15_1591__index.html]

A genome-wide association study in multiple system atrophy — Data Supplement 

# A genome-wide association study in multiple system atrophy

## Data Supplement

**Neurology® data supplements are not copyedited before publication. Published editorials and translations have been copyedited.  
 © 2016 American Academy of Neurology.  
  
 Files in this Data Supplement:**

- Data Supplement - PDF
