# Supplementary material for: A genome-wide association study in multiple system atrophy
Source: Neurology. 2016 Oct 11;87(15):1591–8. doi: 10.1212/WNL.0000000000003221 (PMC5067544; doi:10.1212/WNL.0000000000003221)
Supplement: Data Supplement [file supp_WNL.0000000000003221_SUPPLEMENTAL_MATERIAL.pdf]

**SUPPLEMENTARY MATERIAL**

**Table e-1.** Collection sites

| **MSA Cases** | **N** |
|---|---|
| *Brain Banks* | |
| Queen Square Brain Bank, University College London, London, UK | 137 |
| Institute of Psychiatry Brain Bank, King's College London, London, UK | 13 |
| UK Parkinson's disease tissue bank, Imperial College London, London, UK | 3 |
| Newcastle Brain Tissue Resource, Newcastle University, Newcastle upon Tyne, UK | 6 |
| The Manchester Brain Bank, University of Manchester, Manchester, UK | 2 |
| Jacksonville Brain Bank for Alzheimer's, Parkinson's and Related Disorders, Mayo Clinic Jacksonville, Jacksonville, FL, USA | 64 |
| Miami Brain Bank, Miami, FL, USA | 10 |
| New York Brain Bank, Taub Institute, Columbia University, New York, NY, USA | 11 |
| Center for Neurodegenerative Disease Research, University of Pennsylvania, PA, USA | 34 |
| Harvard Brain Bank, Boston, MA, USA | 2 |
| Human Brain and Spinal Fluid Resource Center, University of California Los Angeles, Los Angeles, CA, USA | 1 |
| Emory University Alzheimer's Disease Research Center Brain Bank, Atlanta, GA, USA | 3 |
| Neurobiobank München, Institut für Neuropathologie, Ludwig-Maximilians-Universität, Munich, Germany | 18 |
| Brain Bank Center Würzburg, Würzburg, Germany | 2 |
| Netherlands Brain Bank, Netherlands Institute for Neuroscience, Amsterdam, Netherlands | 9 |
| Neurological Tissue Bank, University of Barcelona, Hospital Clinic, Barcelona, Spain | 15 |
| *Clinical Centers* | |
| Cardiff University School of Medicine, University Hospital of Wales, Cardiff, UK | 34 |
| National Hospital of Neurology in Neurosurgery, London, UK | 85 |
| National Institute on Neurological Disorders and Stroke, Bethesda, MD, USA | 1 |
| Coriell Cell Repository, Camden, NJ, USA | 24 |
| Hertie Institute for Clinical Brain Research, Tübingen, Germany | 106 |
| Philipps-University of Marburg, Marburg, Germany | 74 |
| Department of Neurology, Christian-Albrechts-Universität Kiel, Kiel, Germany | 5 |
| University Hospital of Bonn Medical Center, Bonn, Germany | 45 |
| Medical University Innsbruck, Innsbruck, Austria | 22 |
| Lund University Hospital, Lund, Denmark | 4 |
| Radboud University Nijmegen Medical Centre, Nijmegen, Netherlands | 59 |
| Hospital Clinic, University of Barcelona, Spain | 20 |
| University Hospital Marqués de Valdecilla, Santander, Spain | 38 |
| University Federico II, Napoli, Italy | 29 |
| Istituti Clinici di Perfezionamento, Milano, Italy | 90 |
| Fondazione IRCCS Istituto Neurologico Carlo Besta, Milan, Italy | 57 |
| Lisbon School of Medicine, Hospital de Santa Maria, Lisbon, Portugal | 7 |

| **Controls** | **N** |
|---|---|
| Wellcome Trust Case Control Consortium (WTCCC) | 936 |
| Hertie Institute for Clinical Brain Research, Tübingen, Germany | 944 |
| US controls, National Institute on Aging, Bethesda, MD, USA | 794 |
| Italian controls, Turin, Italy and Chianti, Italy | 1,190 |

**Table e-2.** Demographic details of MSA cases that passed quality control

| | **UK Cases (n= 239)** | **US Cases (n=129)** | **North. Europe Cases (n=313)** | **South. Europe Cases (n=237)** |
|---|---|---|---|---|
| Male (%) | 62% | 53% | 48% | 46% |
| **Clinically diagnosed cases** | **97** | **19** | **284** | **223** |
| Probable MSA | 13 | 0 | 133 | 9 |
| Possible MSA | 19 | 0 | 32 | 0 |
| NA | 65 | 19 | 119 | 214 |
| **Clinical subtype** | | | | |
| MSA-P | 0 | 1 | 132 | 80 |
| MSA-C | 0 | 0 | 56 | 48 |
| NA | 97 | 18 | 96 | 95 |
| **Definite MSA cases** | **142** | **110** | **29** | **14** |
| SND | 34 | 0 | 0 | 0 |
| OPCA | 27 | 0 | 0 | 0 |
| Mixed | 46 | 0 | 0 | 0 |
| NA | 35 | 110 | 29 | 14 |

Key: SND, striatonigral degeneration; OPCA, olivopontocerebellar atrophy; NA, data not available

**Table e-3.** Subanalysis of pathologically confirmed MSA cases versus healthy controls

| Chr | Position | Marker | Gene (Nearest) | Location | Putative Function | *P* value | OR | Alleles | Allele Freq. | $R^2$ |
|---|---|---|---|---|---|---|---|---|---|---|
| 14 | 89,178,440 | rs4900006 | FOXN3 | intergenic | transcription factor, cell cycle control | 1.10E-07 | 0.24 | C/A | 0.99 | 0.49 |
| 17 | 51,515,165 | chr17:51515165 | ANKFN1 | intergenic | unknown | 1.35E-07 | 0.27 | G/A | 0.96 | 0.54 |
| 8 | 23,659,564 | chr8:23659564 | NKX2-6 | intergenic | embryonic development (heart, pharynx) | 1.17E-06 | 0.28 | C/T | 0.94 | 0.83 |
| 8 | 110,431,637 | rs10108704 | ENY2 | intergenic | component of transcription regulatory histone acetylation complex | 1.48E-06 | 0.59 | G/T | 0.79 | 0.87 |
| 16 | 15,689,134 | chr16:15689134 | NDE1 | intronic | microtubule organization, mitosis and neuronal migration | 1.52E-06 | 0.13 | C/T | 0.99 | 0.69 |
| 6 | 157,454,156 | chr6:157454156 | ARID1B | intronic | transcriptional activation and repression of genes in neuronal development | 3.55E-06 | 0.36 | C/G | 0.91 | 0.60 |
| 6 | 101,308,935 | chr6:101308935 | ASCC3 | intronic | unknown | 3.66E-06 | 0.23 | A/C | 0.97 | 0.57 |
| 8 | 104,768,000 | chr8:104768000 | RIMS2 | intronic | involved in exocytosis | 3.69E-06 | 0.28 | T/C | 0.95 | 0.48 |
| 5 | 165,784,017 | chr5:165784017 | ODZ2 | intergenic | signal transduction | 4.27E-06 | 0.15 | C/T | 0.99 | 0.38 |
| 13 | 75,319,169 | chr13:75319169 | LMO7 | intronic | protein-protein interaction | 5.06E-06 | 0.35 | A/C | 0.98 | 0.60 |
| 2 | 118,166,725 | chr2:118166725 | DDX18 | intergenic | involved in embryogenesis, spermatogenesis, and cellular growth and division | 5.59E-06 | 0.10 | A/C | 0.96 | 0.36 |
| 13 | 71,898,380 | rs1340172 | MIR548I4 | intergenic | microRNA | 6.36E-06 | 5.62 | T/G | 0.98 | 0.54 |
| 18 | 29,577,846 | chr18:29577846 | ASXL3 | exonic | transcription regulation | 6.67E-06 | 0.33 | A/G | 0.95 | 0.96 |
| 2 | 33,556,406 | rs897504 | RASGRP3 | intronic | signal transduction | 7.43E-06 | 0.64 | T/C | 0.95 | 0.95 |
| 17 | 12,760,227 | rs7225504 | ARHGAP44 | intronic | GTPase activator | 7.89E-06 | 0.46 | T/C | 0.97 | 0.56 |
| 16 | 64,525,400 | chr16:64525400 | LOC283867 | intergenic | non-coding RNA | 8.92E-06 | 1.97 | C/G | 0.98 | 0.71 |
| 1 | 54,790,324 | chr1:54790324 | ACOT11 | intronic | lipid metabolism | 9.66E-06 | 0.31 | C/T | 0.99 | 0.52 |

We performed a subanalysis testing only pathologically confirmed MSA cases (n=295) versus healthy controls (n=3,864). This table lists all SNPs with p-values < 1E-6 in this analysis. None of the SNPs tested exceeded the Bonferroni threshold.

**Table e-4.** Candidate SNPs are associated with changes in methylation and expression levels in proximal genomic regions in cerebellar and frontal cortex tissue of healthy controls

| Methylation/ Expression | Brain Region | Gene | SNP | Probe ID | Chr. | Ref./ Alt. | Frequency Effect Allele | RSQR | Effect | SE | *P*-value | FDR Adjusted *P*-value | Distance to Probe (bp) |
|---|---|---|---|---|---|---|---|---|---|---|---|---|---|
| Expression | FCX | ARL17A | rs916888 | ILMN_1698680 | 17 | T,C | 0.2691 | 0.8534 | -0.382 | 0.089 | 1.63E-05 | 0.002745 | 268,888 |
| | CBL | ARL17A | rs916888 | ILMN_1698680 | 17 | T,C | 0.2691 | 0.8534 | -0.286 | 0.088 | 0.001223 | 0.033633 | 268,888 |
| | | RPL19 | rs78523330 | ILMN_1701832 | 17 | A,G | 0.0415 | 0.3740 | -1.06 | 0.309 | 0.000601 | 0.024808 | 252,701 |
| | | PLEKHM1 | rs9303521 | ILMN_1709549 | 17 | T,G | 0.5093 | 0.9849 | 0.28 | 0.072 | 0.000101 | 0.005572 | 291,700 |
| | | PSM83 | rs78523330 | ILMN_1748651 | 17 | A,G | 0.0415 | 0.3740 | -1.265 | 0.309 | 4.26E-05 | 0.003512 | 187,231 |
| | | HS.137078 | rs78523330 | ILMN_1916702 | 17 | A,G | 0.0415 | 0.3740 | -1.071 | 0.318 | 0.000766 | 0.025288 | 20,870 |
| | | LRRC37A4 | rs9303521 | ILMN_2393693 | 17 | T,G | 0.5093 | 0.9849 | 0.603 | 0.075 | 1.13E-15 | 1.86E-15 | 220,662 |
| Methylation | CBL | KIAA1267 | rs916888 | cg19832721 | 17 | T,C | 0.2711 | 0.9202 | 0.396 | 0.103 | 0.000117 | 0.045786 | 613,267 |

Key: Alt, alternative allele; Chr., chromosome; RSQR, imputation quality score; FCX, frontal cortex; CBL, cerebellum; Ref., reference allele; SE, standard error

**Figure e-1.** Study design and analysis workflow

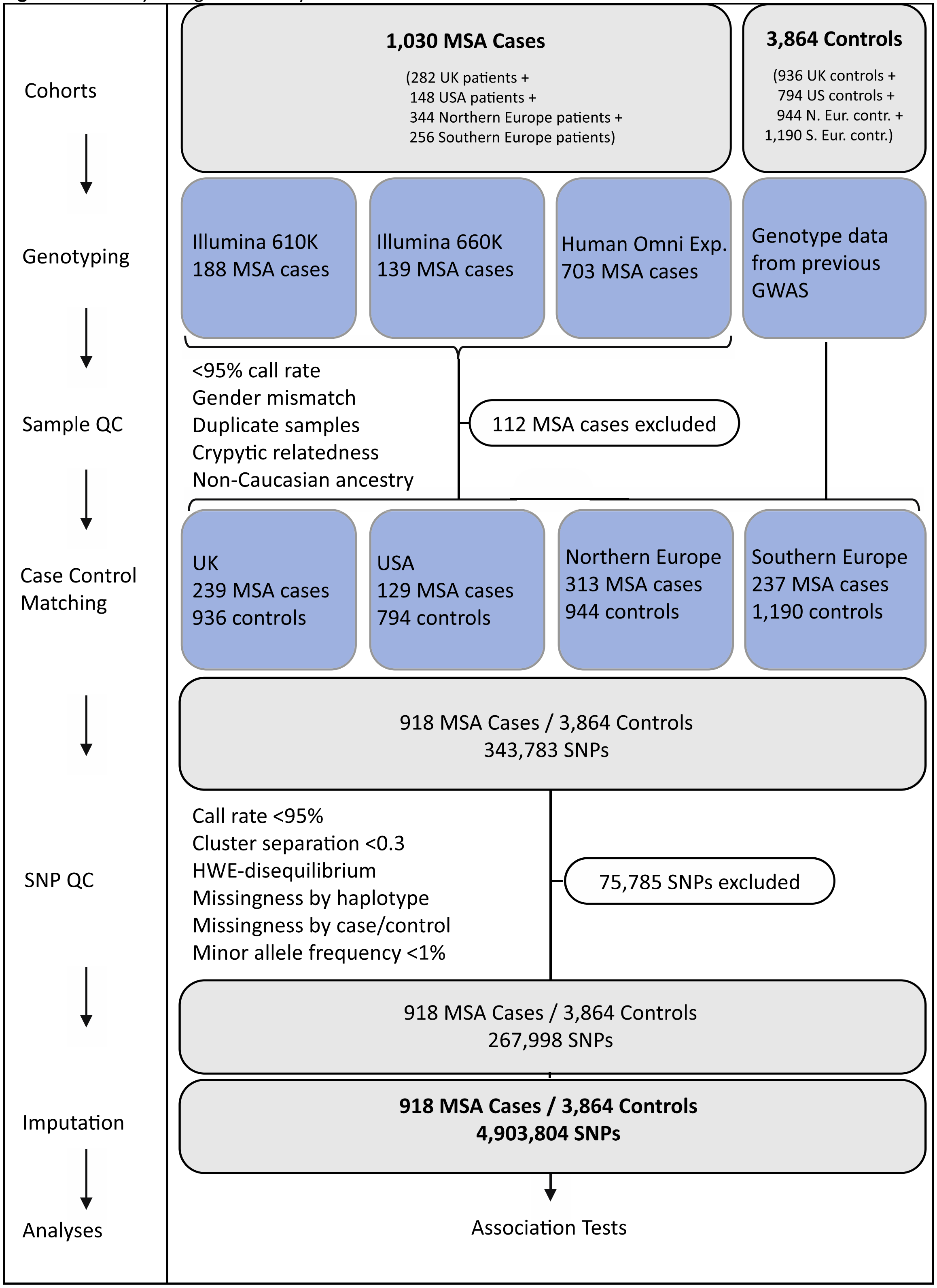

**Figure e-2.** Multidimensional scaling analysis plots for MSA cases and controls before and after removal of outliers

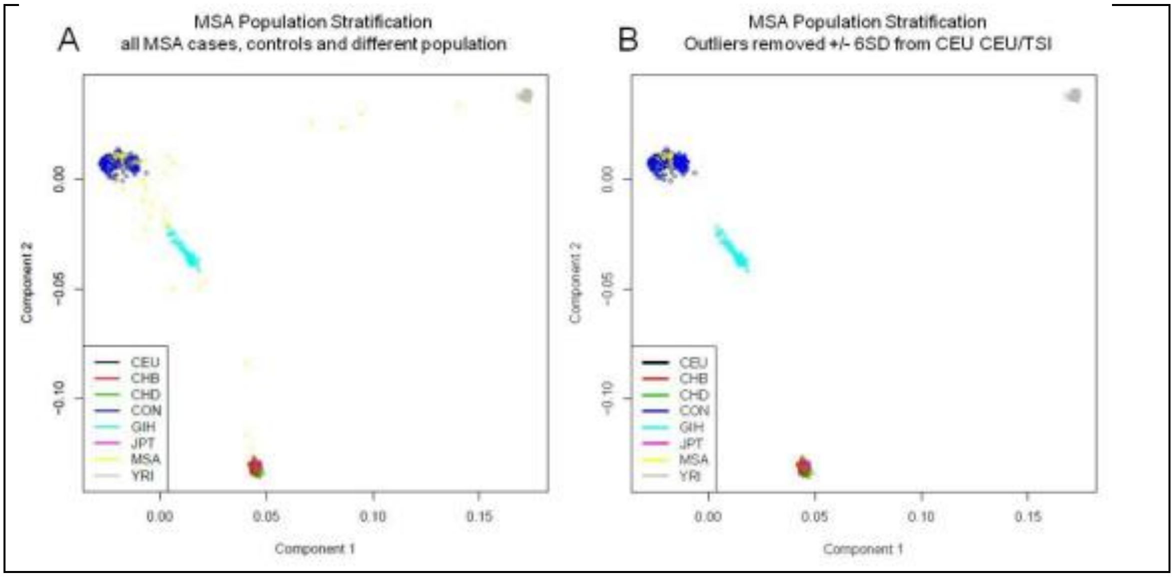


MSA cases and controls used in the GWAS were clustered together with data from different populations. Cases and controls cluster together with the European control populations (A). Samples that deviated from the European control population (CEU/TSI) by more than six standard deviations were excluded as likely to be not of European ancestry (B). (CEU = Utah residents with Northern and Western European ancestry; CHB = Han Chinese in Beijing; CHD = Chinese in Metropolitan Denver; CON = Controls in this study; GIH = Gujarati Indians in Houston, Texas; JPT = Japanese in Tokyo; MSA = MSA GWAS cases; YRI = Yoruba in Ibadan, Nigeria).

**Figure e-3.** Power simulation

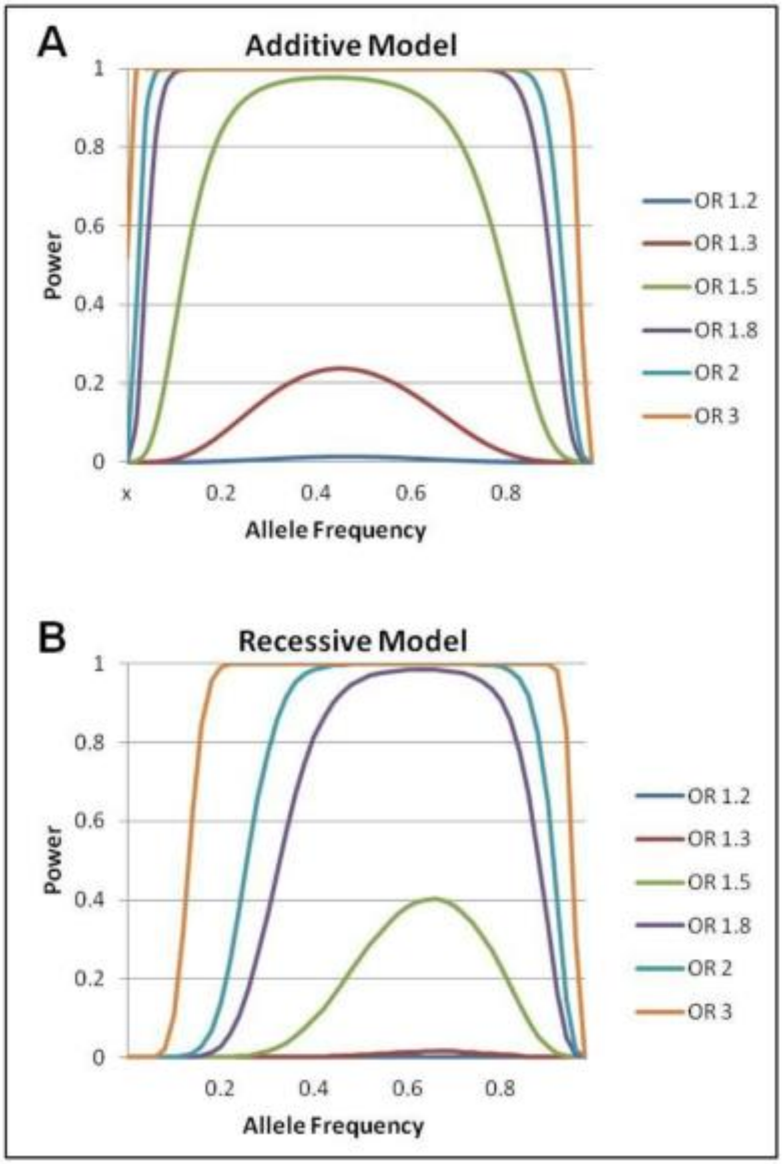

**Figure e-4.** Regional association plots

24 regional association plots for SNPs with the lowest p-values +/- 1 Mb surrounding this SNP are shown. The r2 pattern is based on the SNP with the lowest p-value per locus

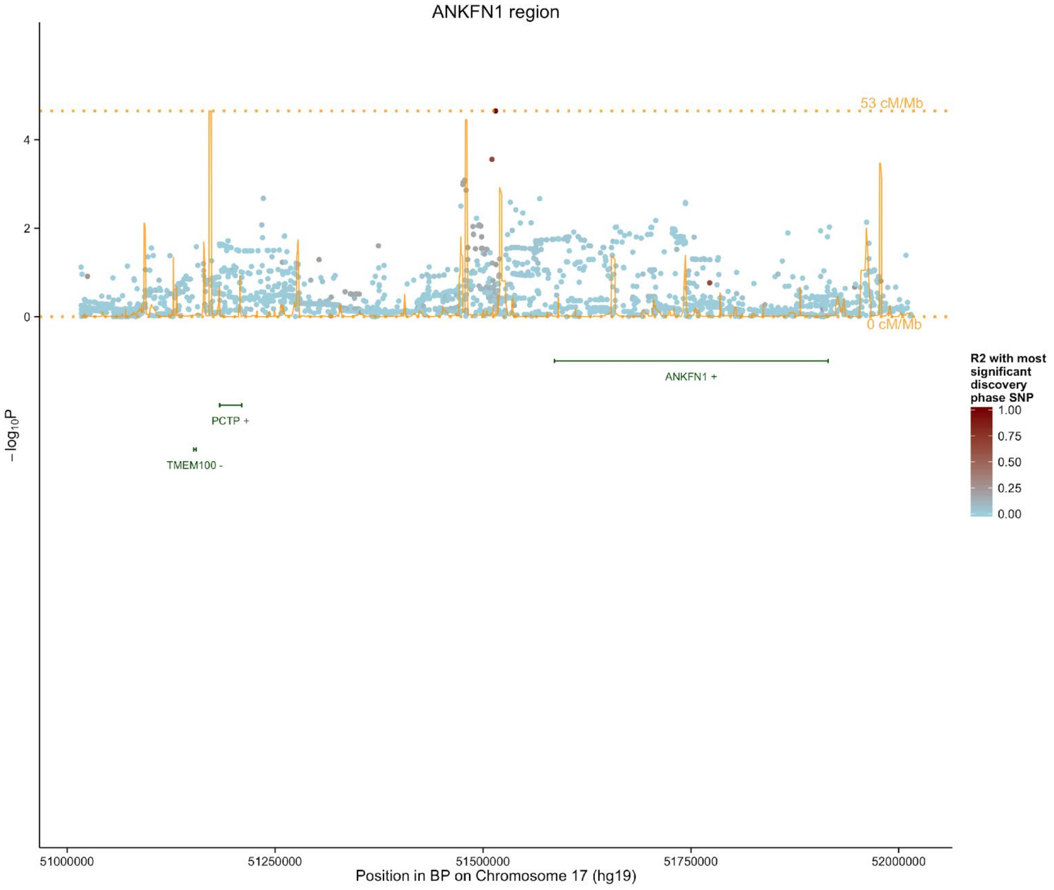
ANKFN1 region
53 cM/Mb
0 cM/Mb
4
2
0
−log10P
ANKFN1 +
PCTP +
TMEM100 -
51000000
51250000
51500000
51750000
52000000
Position in BP on Chromosome 17 (hg19)
R2 with most significant discovery phase SNP
1.00
0.75
0.50
0.25
0.00

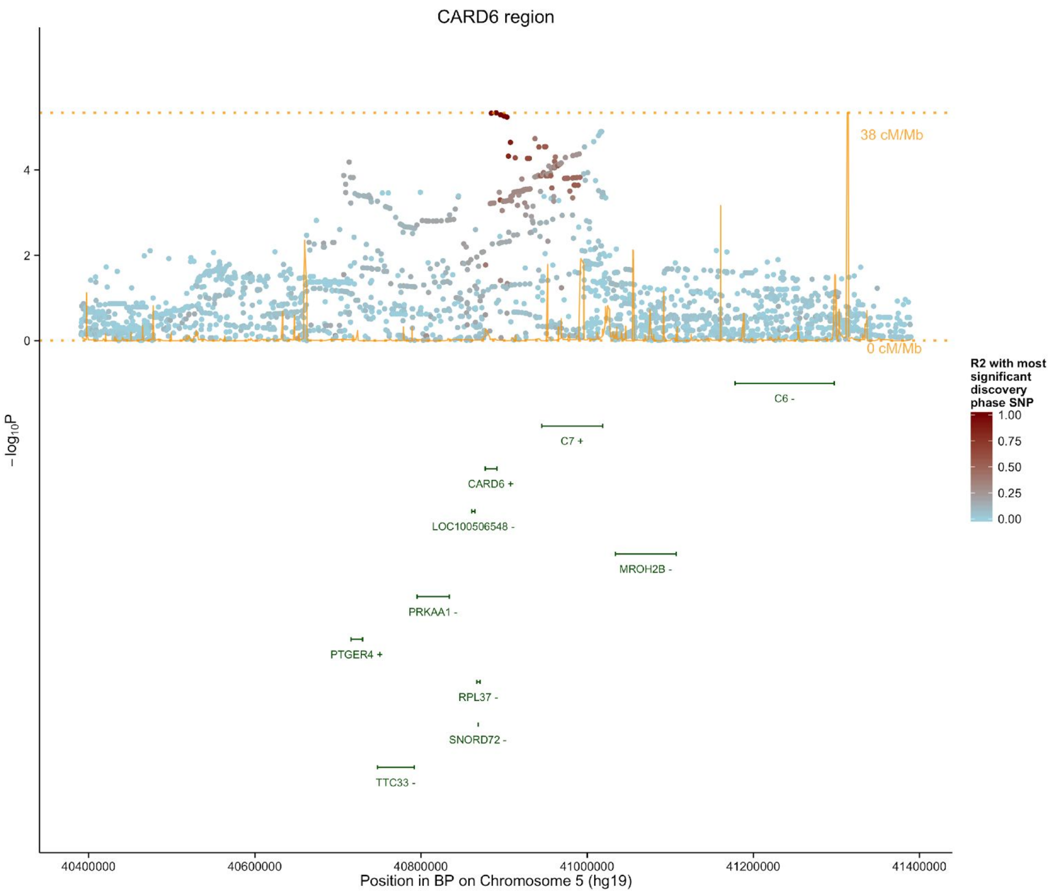
CARD6 region
38 cM/Mb
0 cM/Mb
4
2
0
$-\log_{10}P$
C6 -
C7 +
CARD6 +
LOC100506548 -
MROH2B -
PRKAA1 -
PTGER4 +
RPL37 -
SNORD72 -
TTC33 -
40400000
40600000
40800000
41000000
41200000
41400000
Position in BP on Chromosome 5 (hg19)
R2 with most significant discovery phase SNP
1.00
0.75
0.50
0.25
0.00

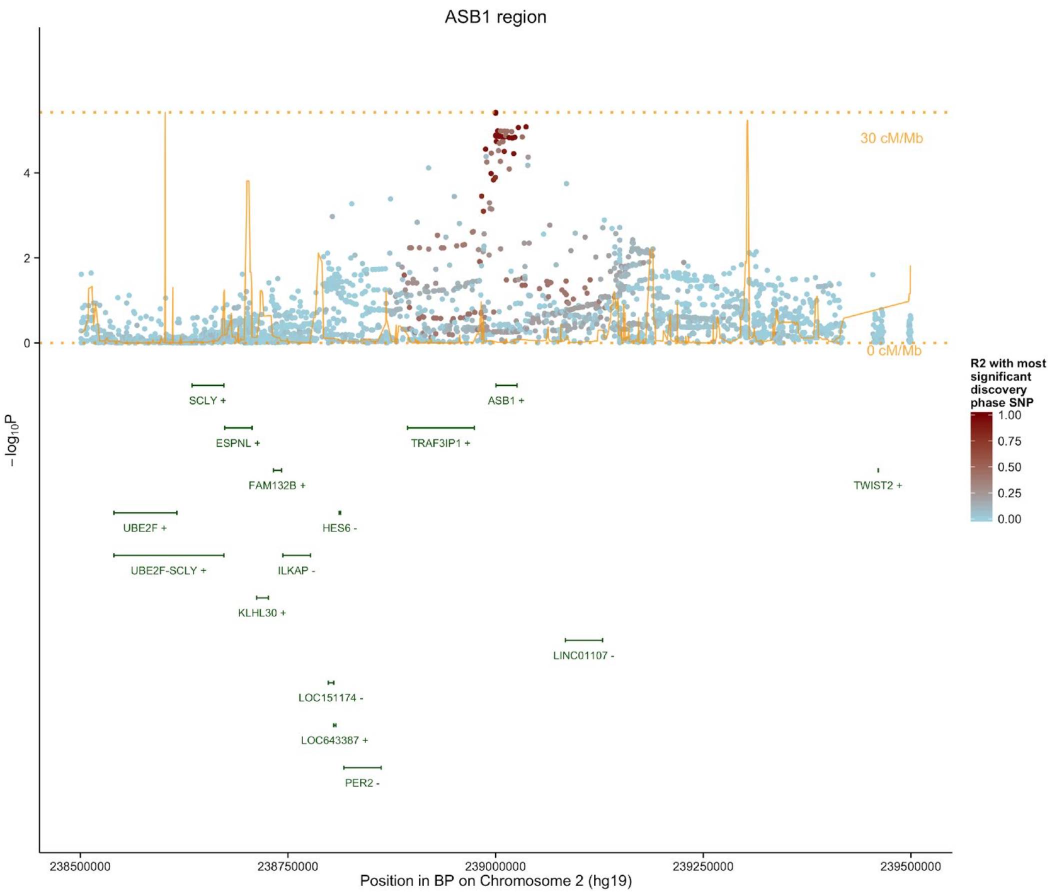
ASB1 region
30 cM/Mb
0 cM/Mb
4
2
0
− log10P
SCLY +
ASB1 +
ESPNL +
TRAF3IP1 +
FAM132B +
TWIST2 +
UBE2F +
HES6 -
UBE2F-SCLY +
ILKAP -
KLHL30 +
LINC01107 -
LOC151174 -
LOC643387 +
PER2 -
238500000
238750000
239000000
239250000
239500000
Position in BP on Chromosome 2 (hg19)
R2 with most significant discovery phase SNP
1.00
0.75
0.50
0.25
0.00

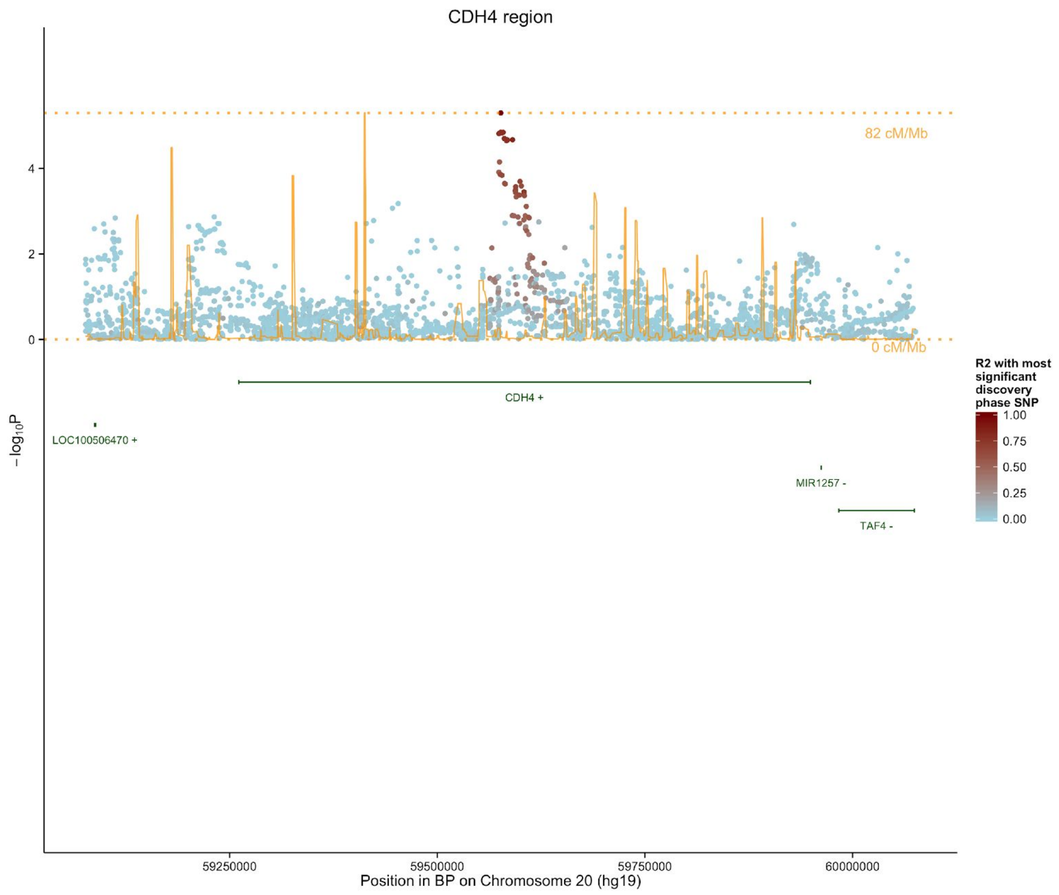
CDH4 region
82 cM/Mb
0 cM/Mb
4
2
0
−log10P
CDH4 +
LOC100506470 +
MIR1257 -
TAF4 -
R2 with most significant discovery phase SNP
1.00
0.75
0.50
0.25
0.00
59250000
59500000
59750000
60000000
Position in BP on Chromosome 20 (hg19)

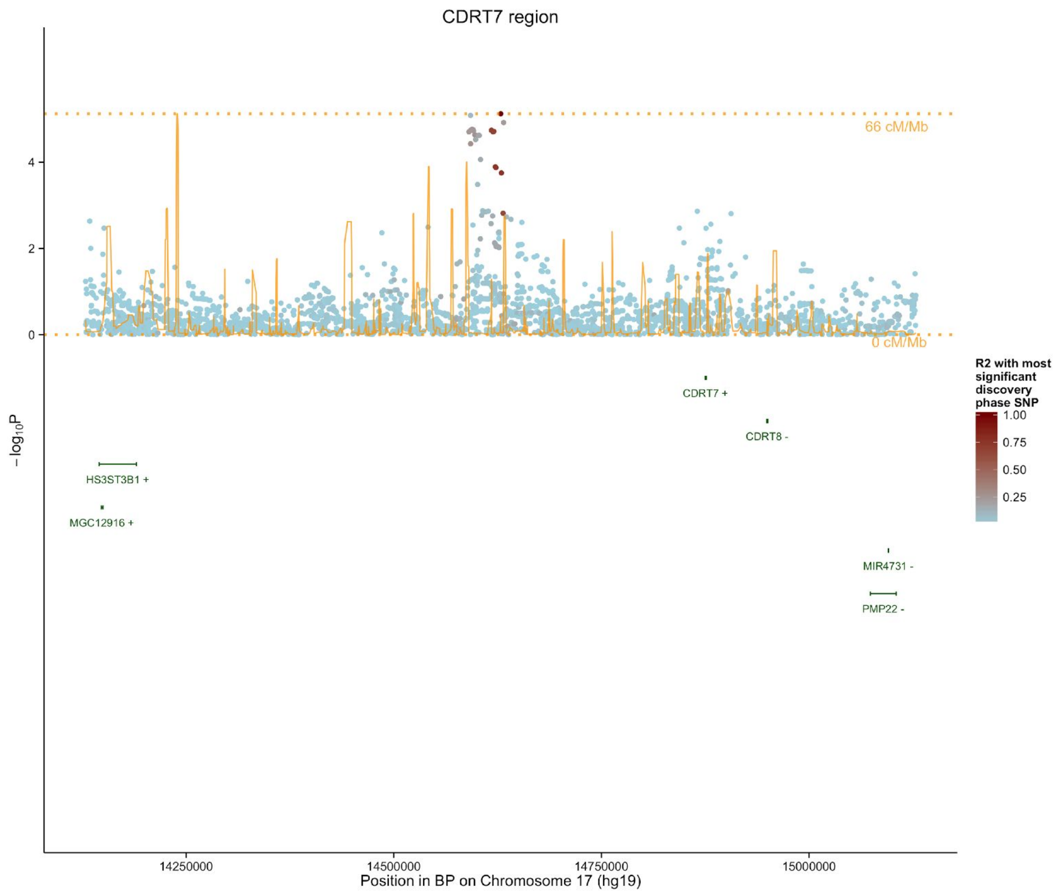
CDRT7 region
66 cM/Mb
0 cM/Mb
4
2
0
−log10P
CDRT7 +
CDRT8 -
HS3ST3B1 +
MGC12916 +
MIR4731 -
PMP22 -
14250000
14500000
14750000
15000000
Position in BP on Chromosome 17 (hg19)
R2 with most significant discovery phase SNP
1.00
0.75
0.50
0.25

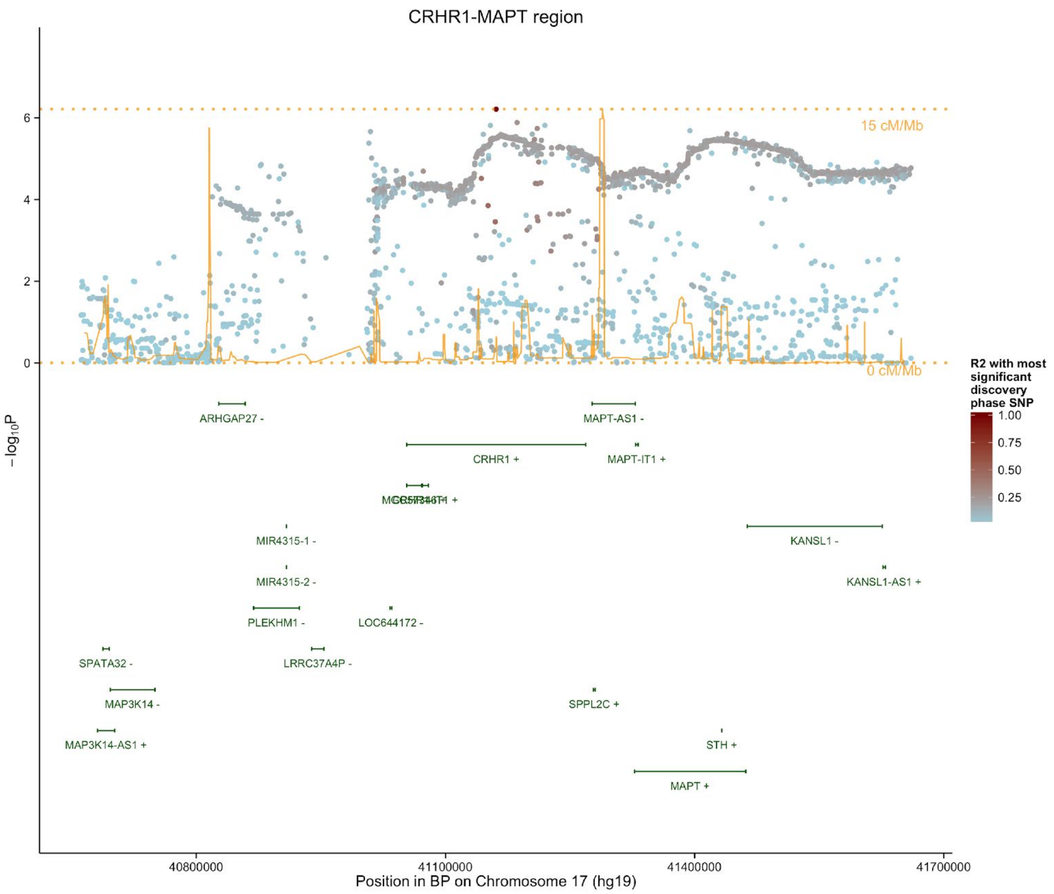
CRHR1-MAPT region
15 cM/Mb
0 cM/Mb
−log10P
R2 with most significant discovery phase SNP
1.00
0.75
0.50
0.25
ARHGAP27 -
MAPT-AS1 -
CRHR1 +
MAPT-IT1 +
MIR4315-1 -
KANSL1 -
MIR4315-2 -
KANSL1-AS1 +
PLEKHM1 -
LOC644172 -
SPATA32 -
LRRC37A4P -
MAP3K14 -
SPPL2C +
MAP3K14-AS1 +
STH +
MAPT +
40800000
41100000
41400000
41700000
Position in BP on Chromosome 17 (hg19)

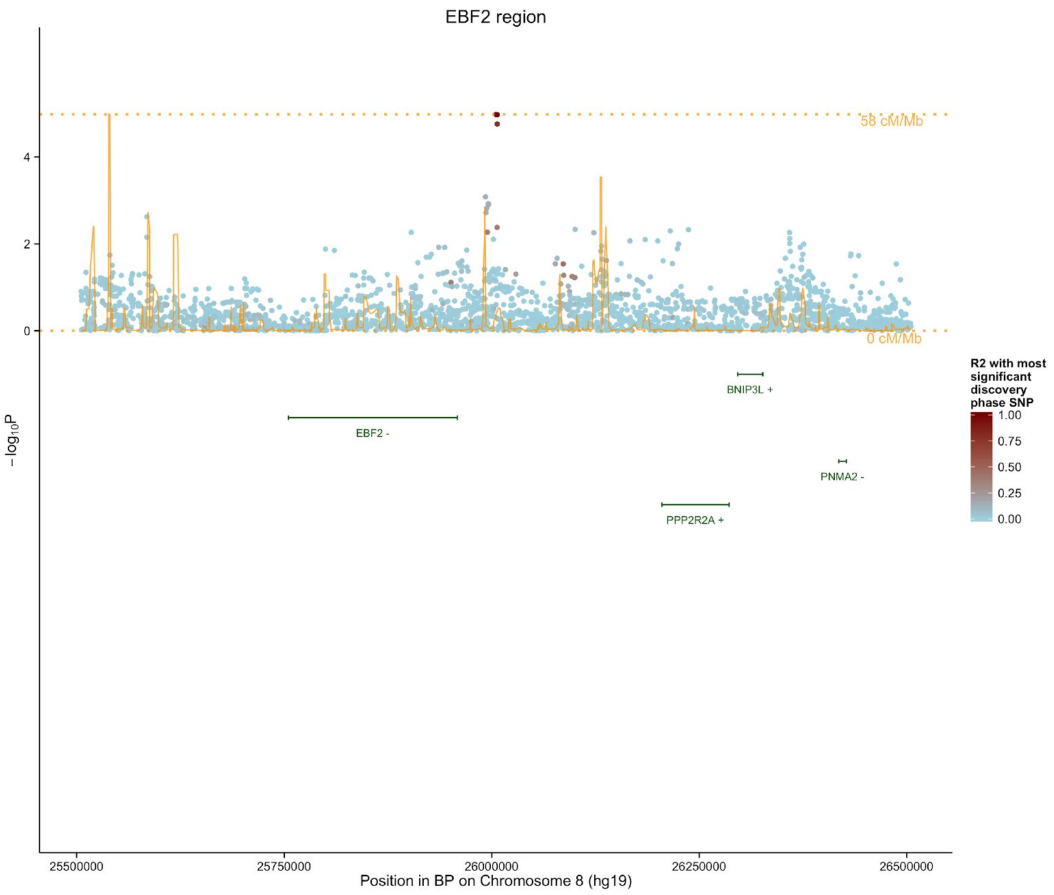
EBF2 region
58 cM/Mb
0 cM/Mb
4
2
0
$-\log_{10}P$
BNIP3L +
EBF2 -
PNMA2 -
PPP2R2A +
R2 with most significant discovery phase SNP
1.00
0.75
0.50
0.25
0.00
25500000
25750000
26000000
26250000
26500000
Position in BP on Chromosome 8 (hg19)

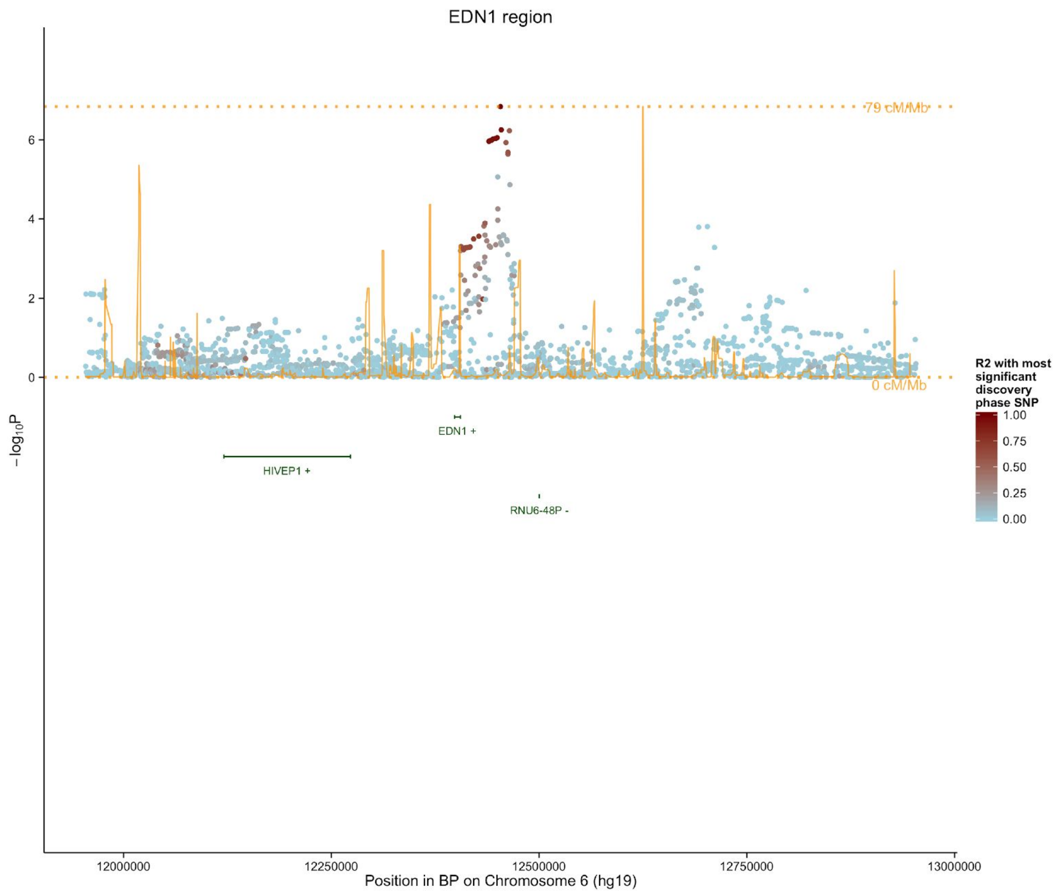
EDN1 region
79 cM/Mb
0 cM/Mb
6
4
2
0
$-\log_{10}P$
EDN1 +
HIVEP1 +
RNU6-48P -
12000000
12250000
12500000
12750000
13000000
Position in BP on Chromosome 6 (hg19)
R2 with most significant discovery phase SNP
1.00
0.75
0.50
0.25
0.00

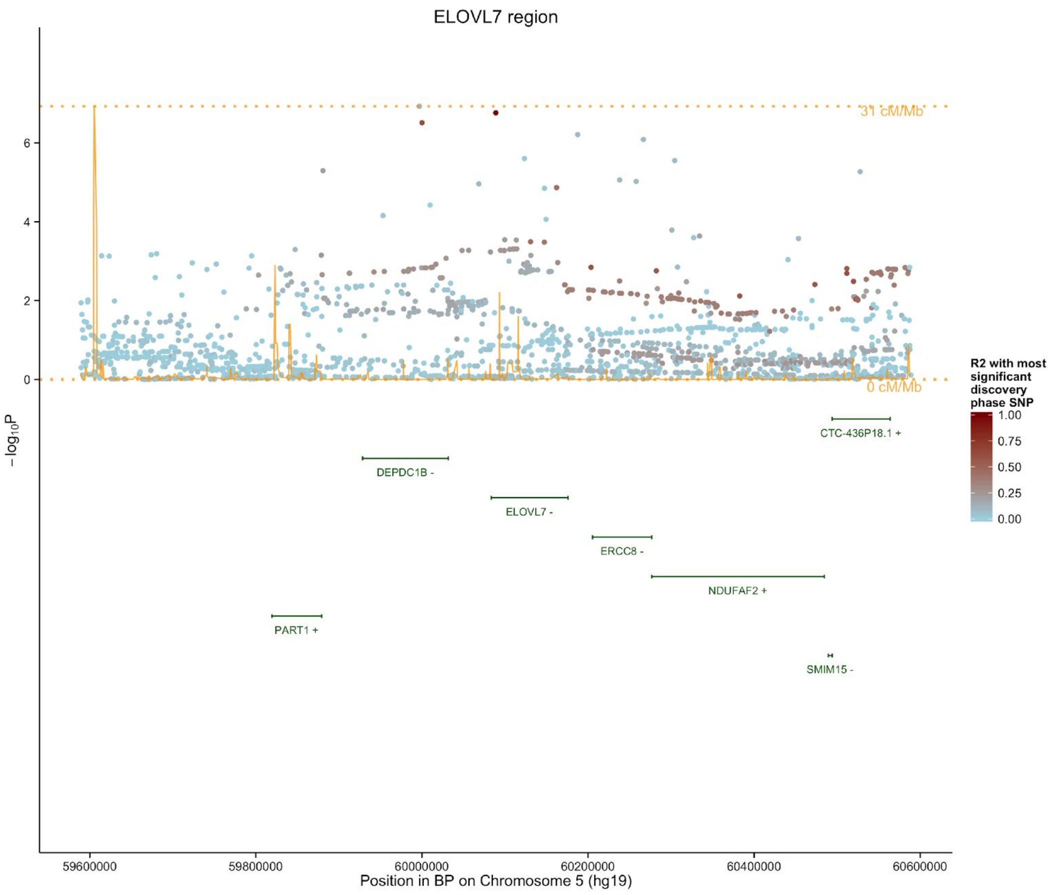
ELOVL7 region
31 cM/Mb
0 cM/Mb
6
4
2
0
$-\log_{10}P$
R2 with most significant discovery phase SNP
1.00
0.75
0.50
0.25
0.00
CTC-436P18.1 +
DEPDC1B -
ELOVL7 -
ERCC8 -
NDUFAF2 +
PART1 +
SMIM15 -
59600000
59800000
60000000
60200000
60400000
60600000
Position in BP on Chromosome 5 (hg19)

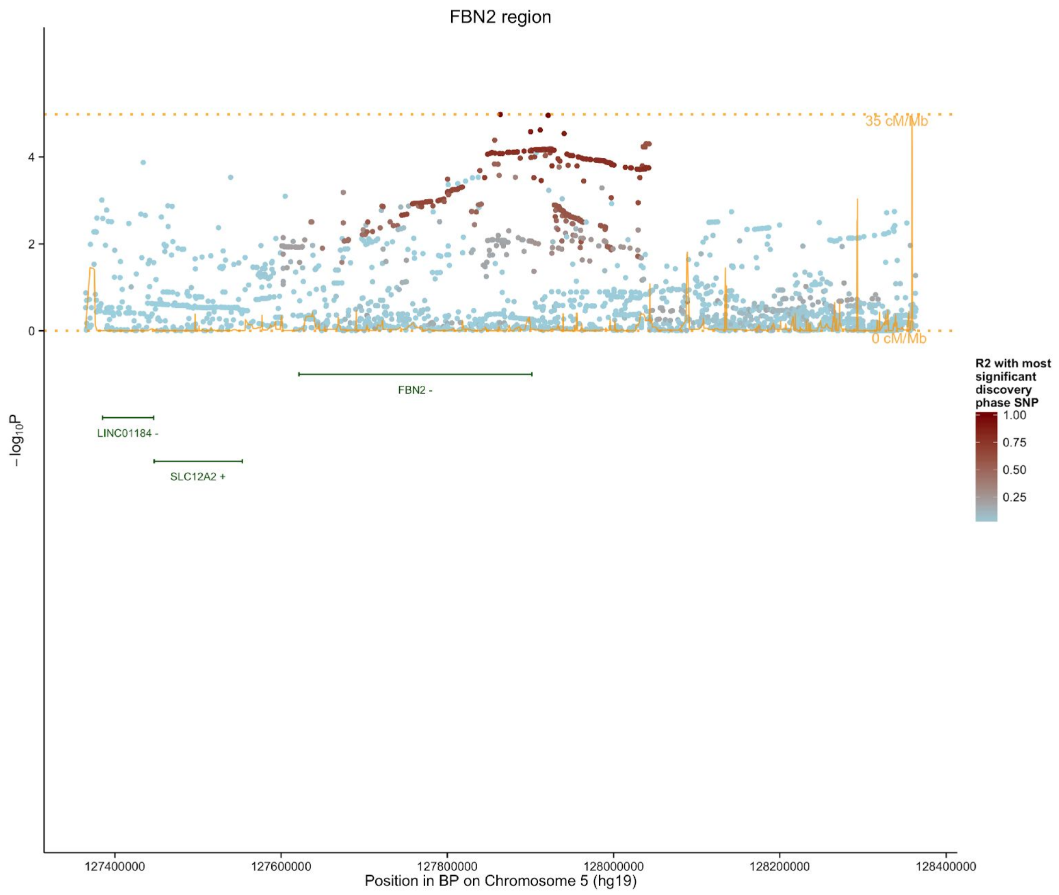

FBN2 region
35 cM/Mb
0 cM/Mb
4
2
0
−log10P
FBN2 -
LINC01184 -
SLC12A2 +
R2 with most significant discovery phase SNP
1.00
0.75
0.50
0.25
127400000
127600000
127800000
128000000
128200000
128400000
Position in BP on Chromosome 5 (hg19)

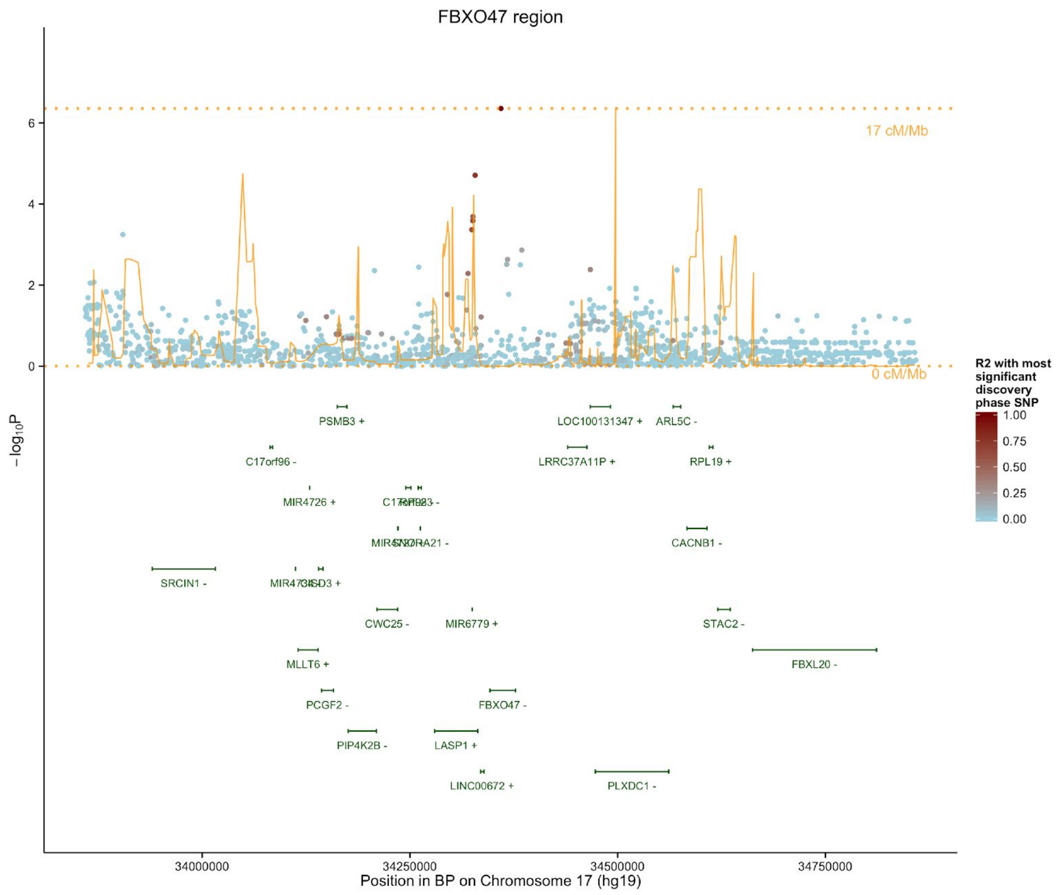

FBXO47 region
17 cM/Mb
0 cM/Mb
6
4
2
0
$-\log_{10}P$
PSMB3 +
LOC100131347 +
ARL5C -
C17orf96 -
LRRC37A11P +
RPL19 +
MIR4726 +
CACNB1 -
SRCIN1 -
CWC25 -
MIR6779 +
STAC2 -
MLLT6 +
FBXL20 -
PCGF2 -
FBXO47 -
PIP4K2B -
LASP1 +
LINC00672 +
PLXDC1 -
34000000
34250000
34500000
34750000
Position in BP on Chromosome 17 (hg19)
R2 with most significant discovery phase SNP
1.00
0.75
0.50
0.25
0.00

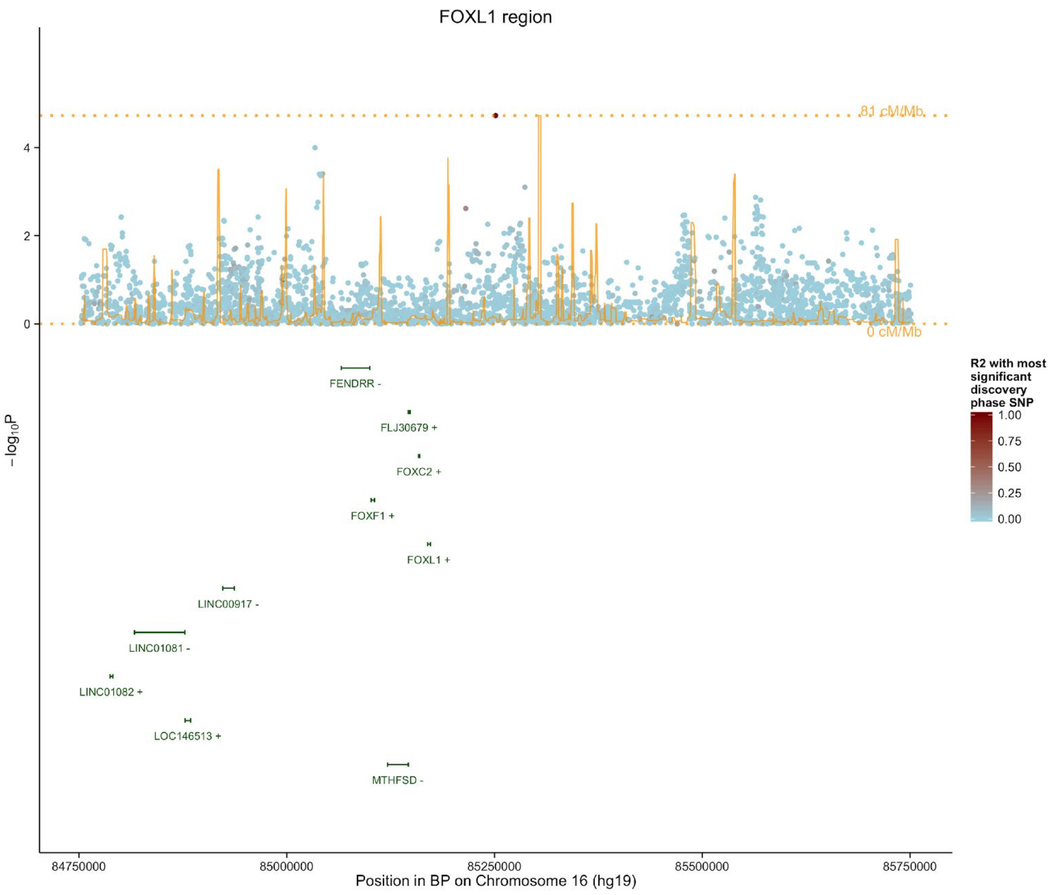

FOXL1 region
81 cM/Mb
0 cM/Mb
4
2
0
$-\log_{10}P$
FENDRR -
FLJ30679 +
FOXC2 +
FOXF1 +
FOXL1 +
LINC00917 -
LINC01081 -
LINC01082 +
LOC146513 +
MTHFSD -
84750000
85000000
85250000
85500000
85750000
Position in BP on Chromosome 16 (hg19)
R2 with most significant discovery phase SNP
1.00
0.75
0.50
0.25
0.00

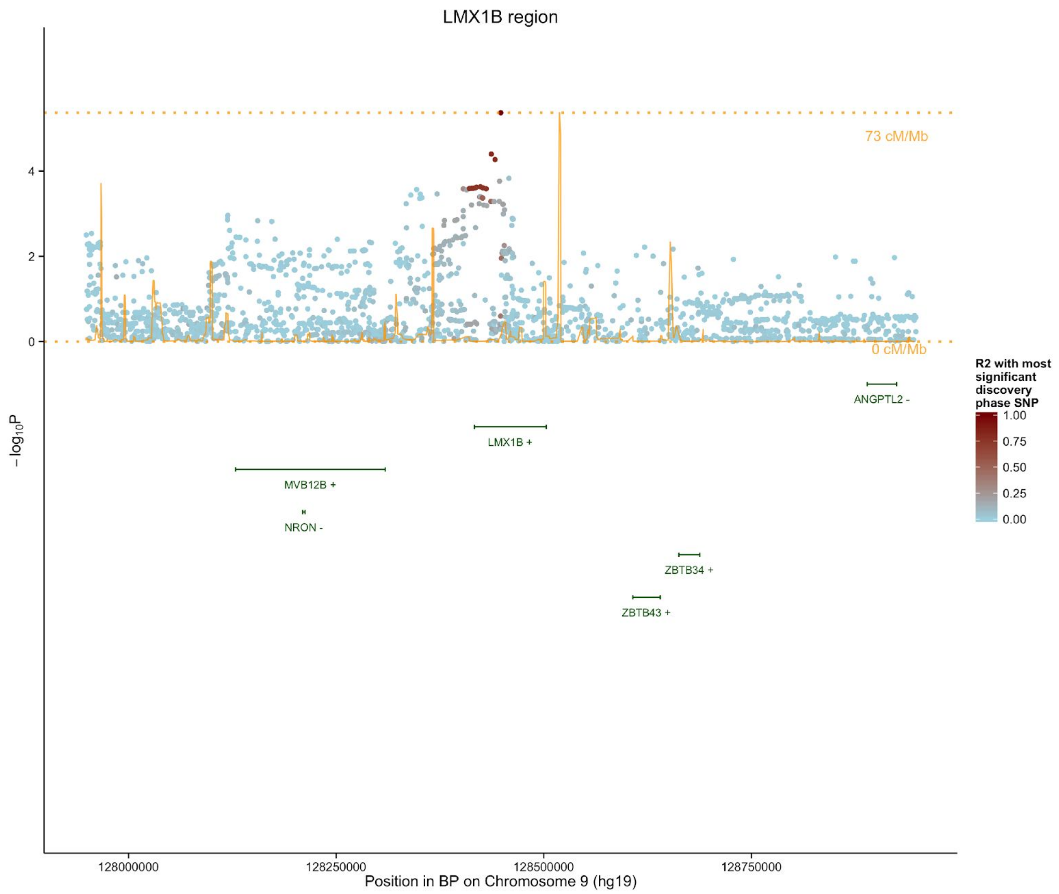
LMX1B region
73 cM/Mb
0 cM/Mb
4
2
0
−log10P
R2 with most significant discovery phase SNP
1.00
0.75
0.50
0.25
0.00
ANGPTL2 -
LMX1B +
MVB12B +
NRON -
ZBTB34 +
ZBTB43 +
128000000
128250000
128500000
128750000
Position in BP on Chromosome 9 (hg19)

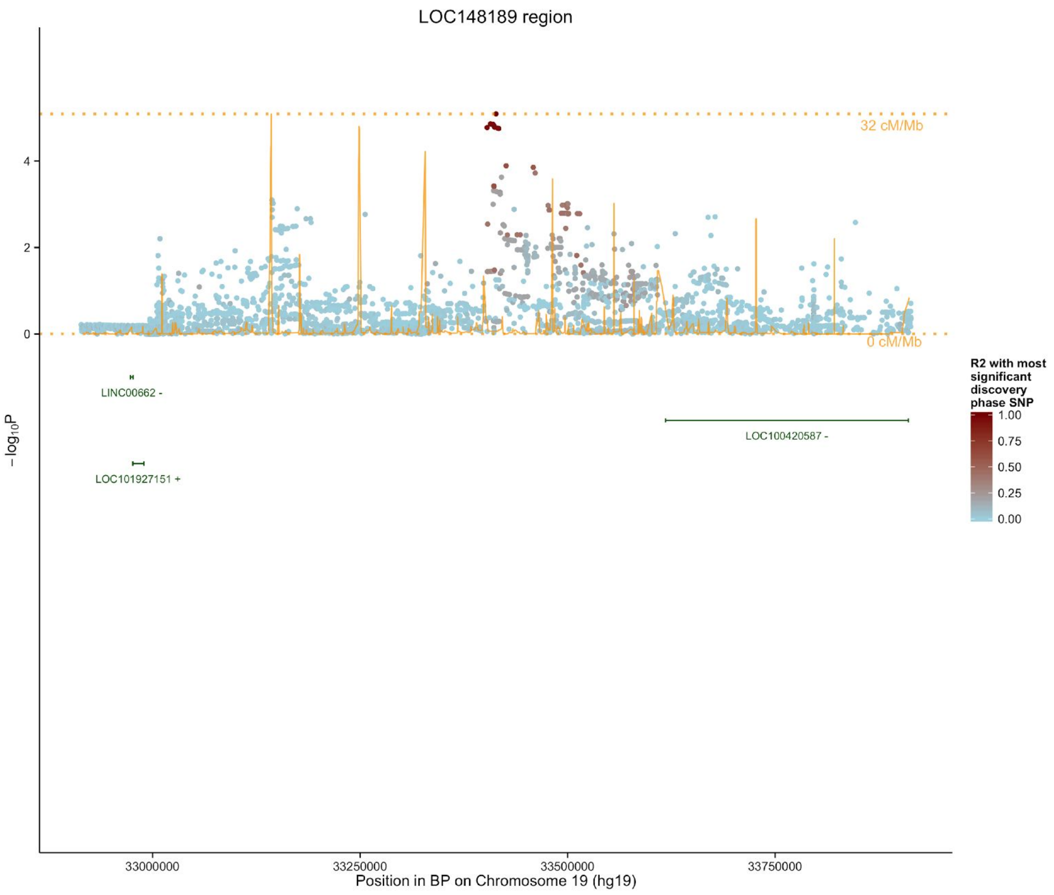

LOC148189 region
32 cM/Mb
0 cM/Mb
4
2
0
−log10P
LINC00662 -
LOC101927151 +
LOC100420587 -
R2 with most significant discovery phase SNP
1.00
0.75
0.50
0.25
0.00
33000000
33250000
33500000
33750000
Position in BP on Chromosome 19 (hg19)

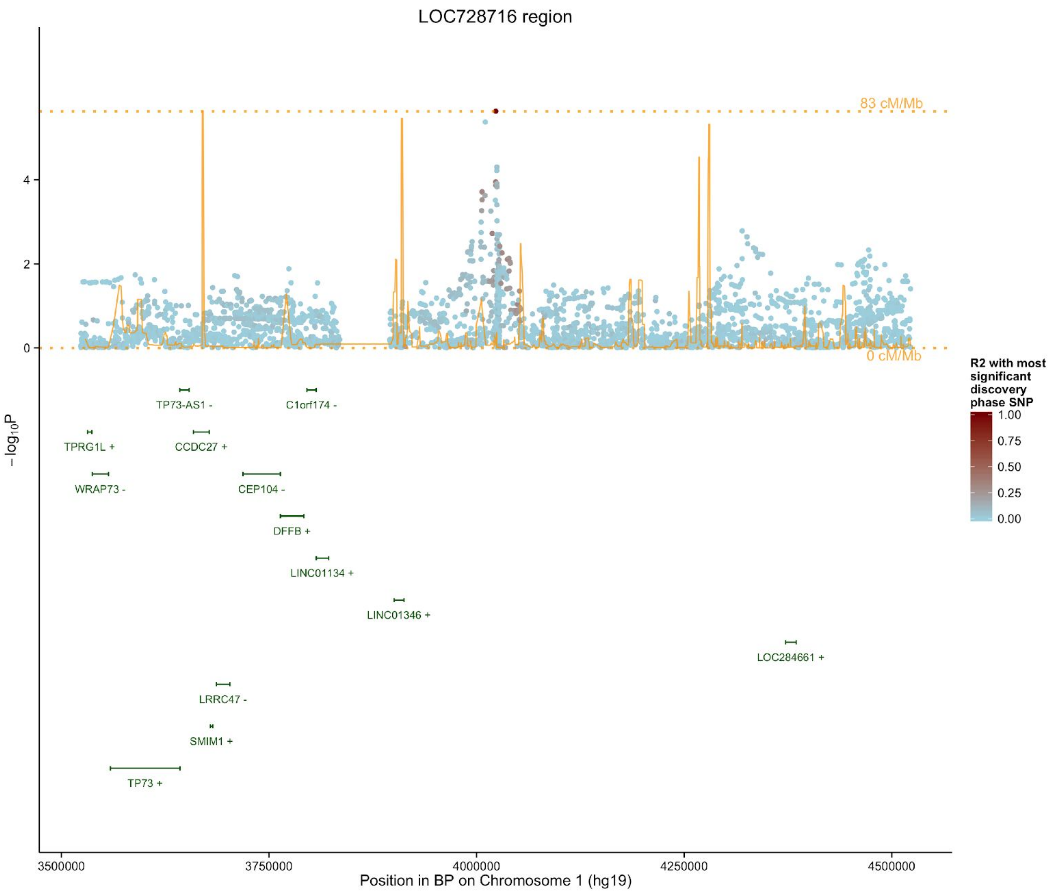

LOC728716 region
83 cM/Mb
0 cM/Mb
4
2
0
−log10P
TP73-AS1 -
C1orf174 -
TPRG1L +
CCDC27 +
WRAP73 -
CEP104 -
DFFB +
LINC01134 +
LINC01346 +
LOC284661 +
LRRC47 -
SMIM1 +
TP73 +
3500000
3750000
4000000
4250000
4500000
Position in BP on Chromosome 1 (hg19)
R2 with most significant discovery phase SNP
1.00
0.75
0.50
0.25
0.00

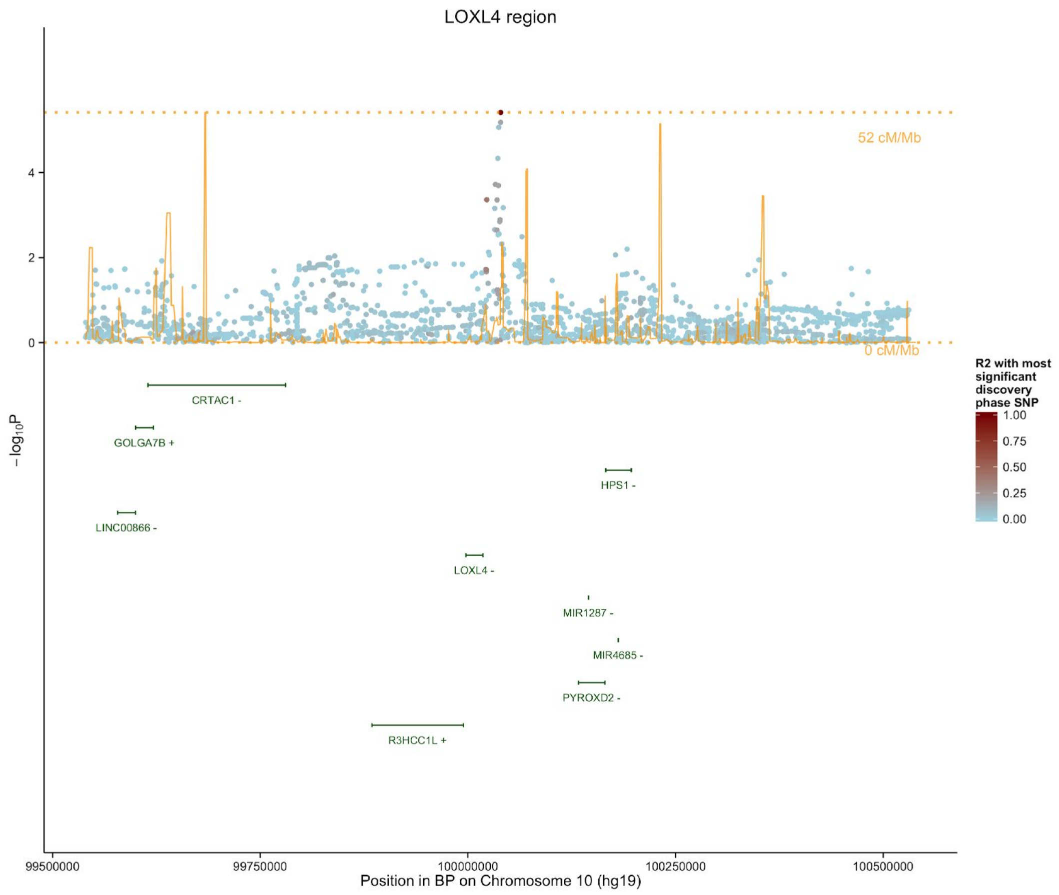
LOXL4 region
52 cM/Mb
0 cM/Mb
4
2
0
−log10P
CRTAC1 -
GOLGA7B +
LINC00866 -
HPS1 -
LOXL4 -
MIR1287 -
MIR4685 -
PYROXD2 -
R3HCC1L +
99500000
99750000
100000000
100250000
100500000
Position in BP on Chromosome 10 (hg19)
R2 with most significant discovery phase SNP
1.00
0.75
0.50
0.25
0.00

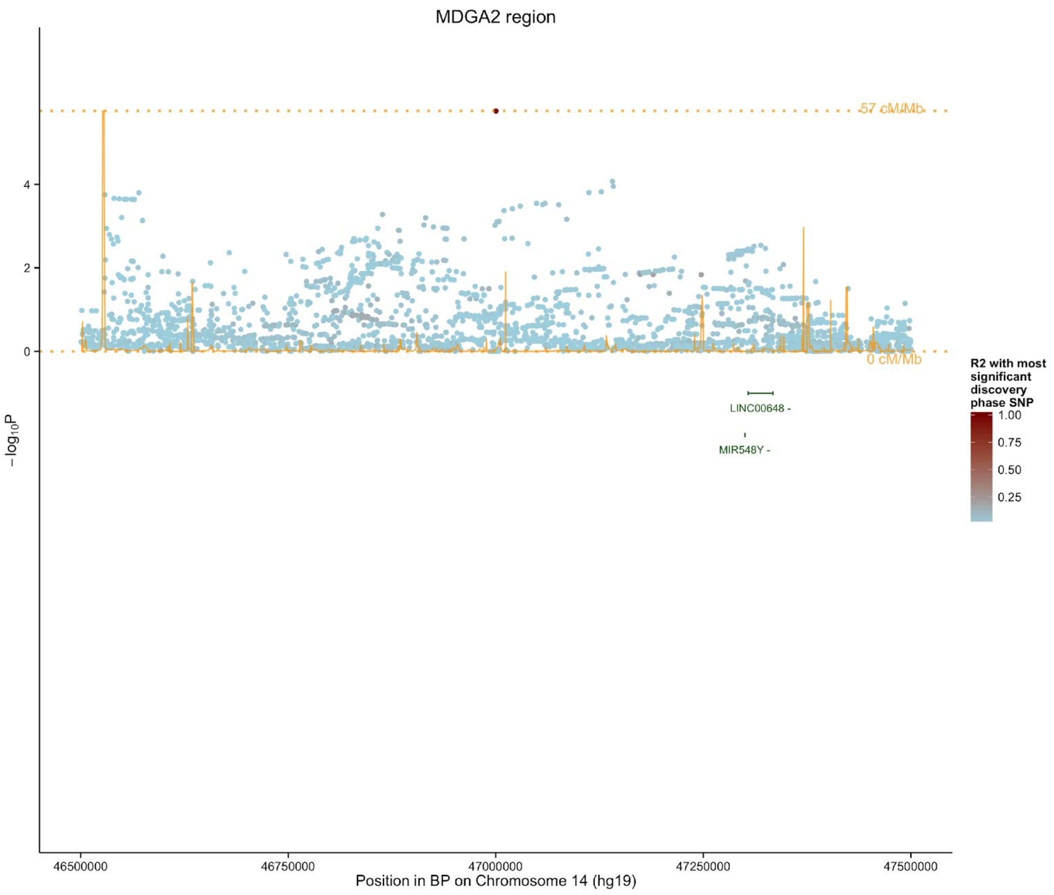

MDGA2 region
57 cM/Mb
0 cM/Mb
4
2
0
$-\log_{10}P$
LINC00648 -
MIR548Y -
R2 with most significant discovery phase SNP
1.00
0.75
0.50
0.25
46500000
46750000
47000000
47250000
47500000
Position in BP on Chromosome 14 (hg19)

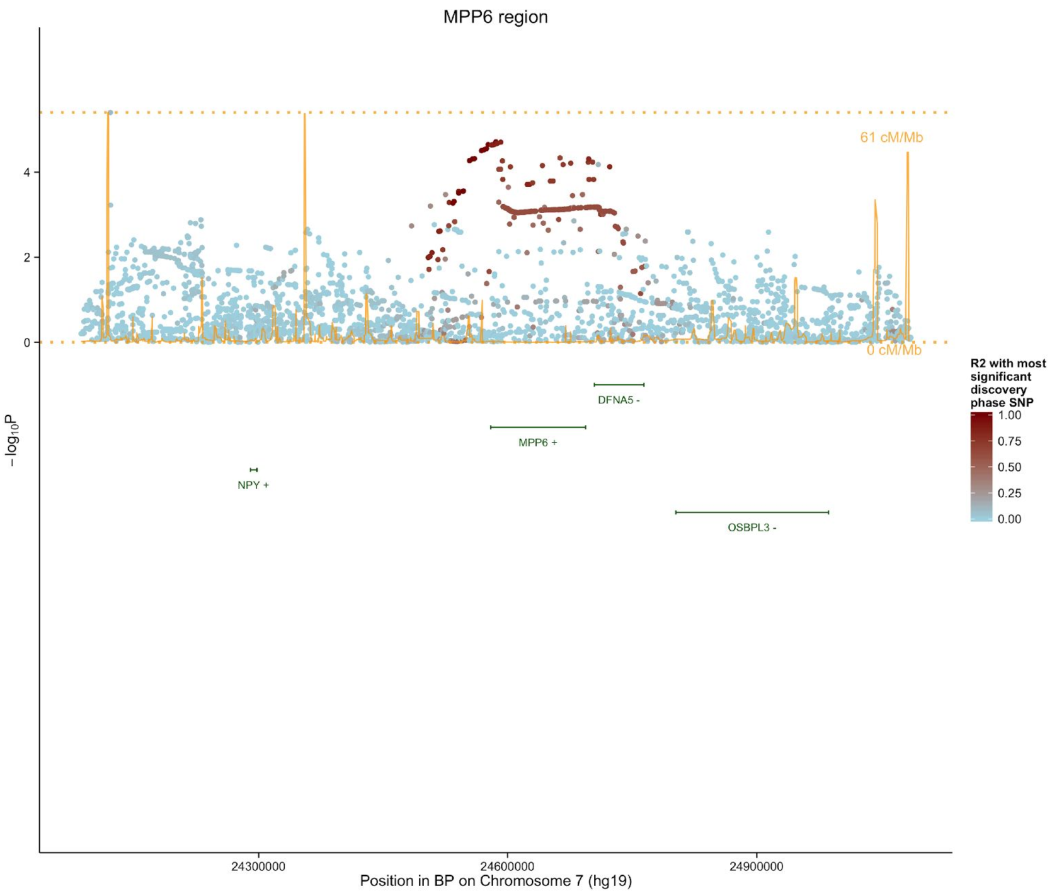
MPP6 region
61 cM/Mb
0 cM/Mb
4
2
0
$-\log_{10}P$
DFNA5 -
MPP6 +
NPY +
OSBPL3 -
24300000
24600000
24900000
Position in BP on Chromosome 7 (hg19)
R2 with most significant discovery phase SNP
1.00
0.75
0.50
0.25
0.00

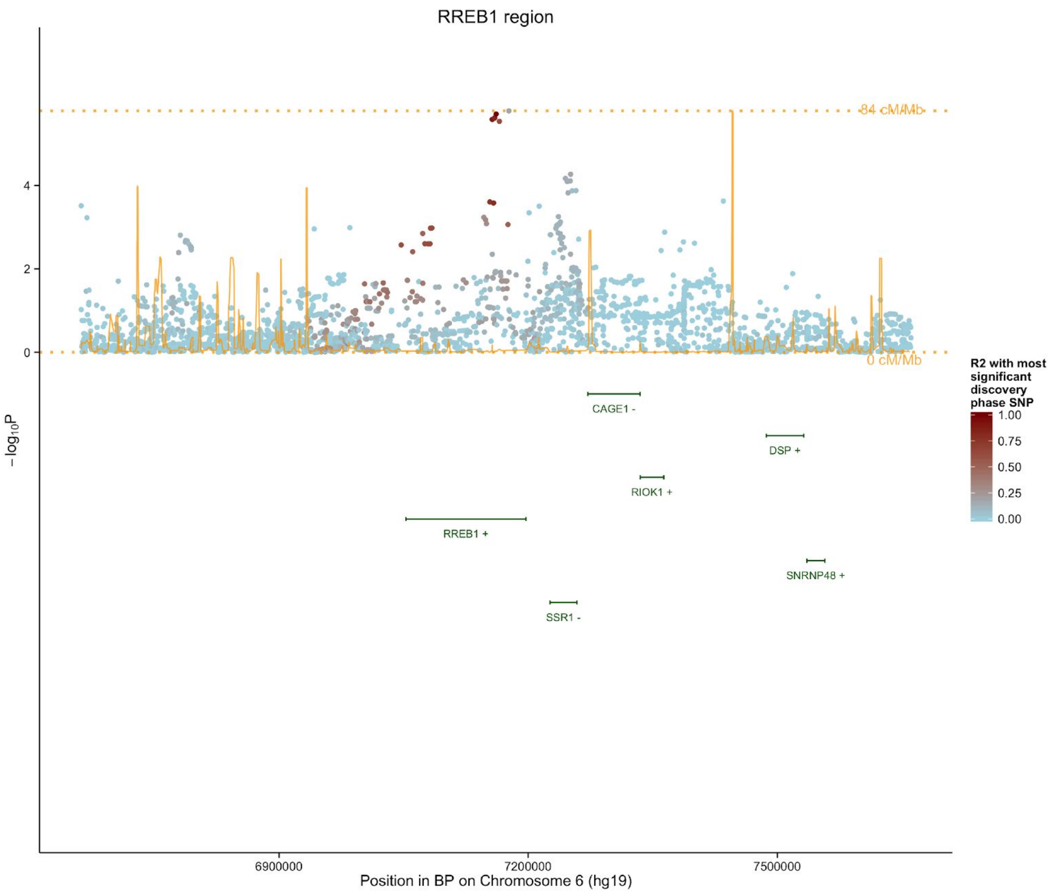

RREB1 region
84 cM/Mb
0 cM/Mb
4
2
0
−log10P
CAGE1 -
DSP +
RIOK1 +
RREB1 +
SNRNP48 +
SSR1 -
R2 with most significant discovery phase SNP
1.00
0.75
0.50
0.25
0.00
6900000
7200000
7500000
Position in BP on Chromosome 6 (hg19)

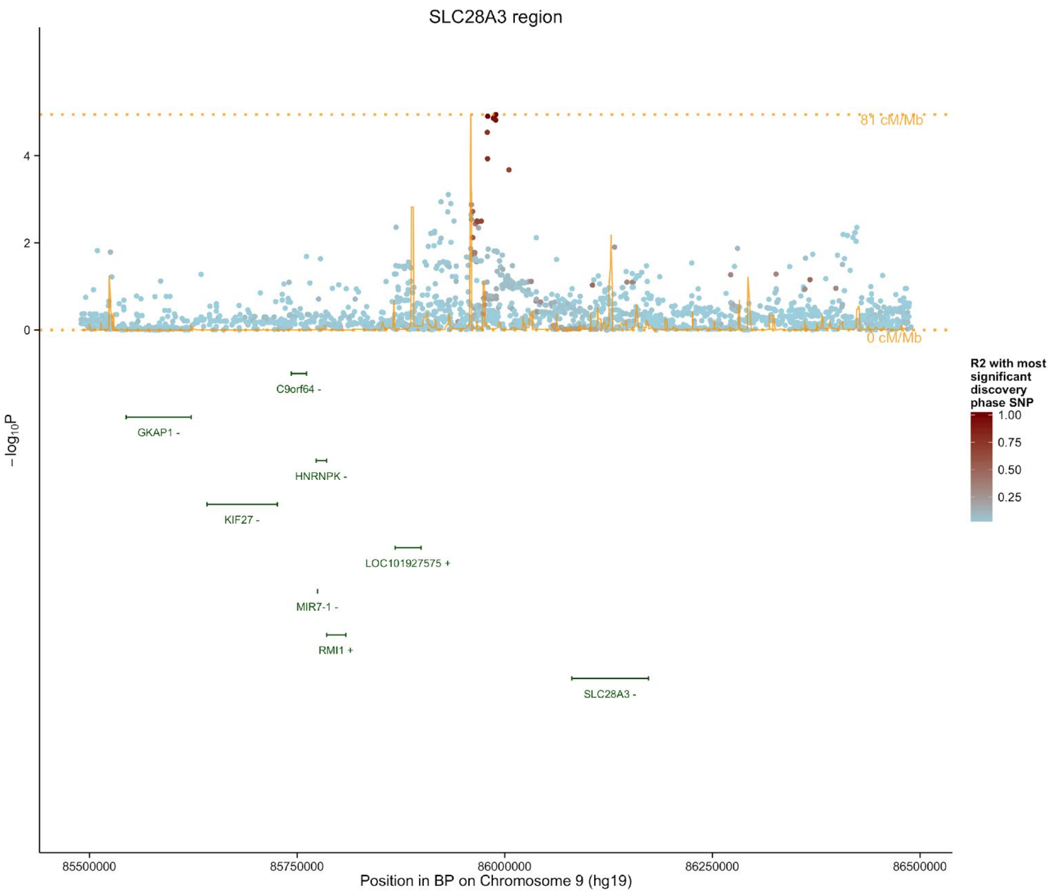
SLC28A3 region
81 cM/Mb
0 cM/Mb
4
2
0
−log10P
C9orf64 -
GKAP1 -
HNRNPK -
KIF27 -
LOC101927575 +
MIR7-1 -
RMI1 +
SLC28A3 -
85500000
85750000
86000000
86250000
86500000
Position in BP on Chromosome 9 (hg19)
R2 with most significant discovery phase SNP
1.00
0.75
0.50
0.25

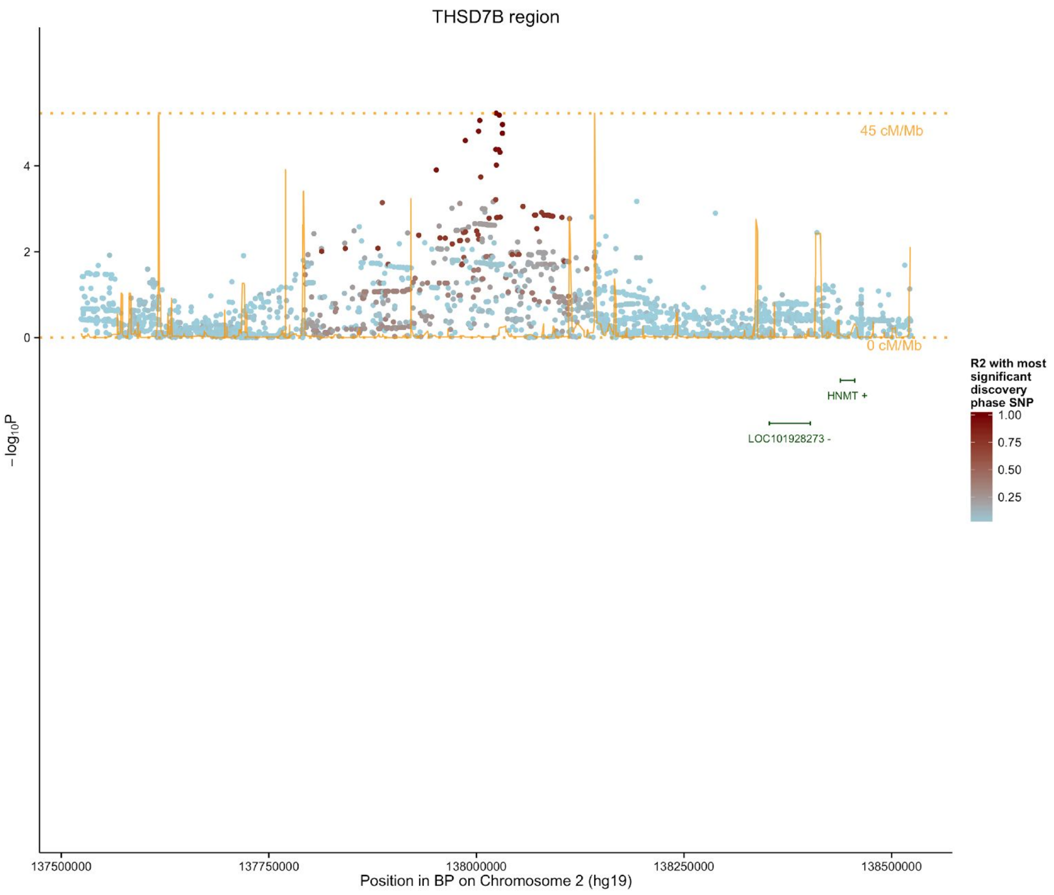
THSD7B region
45 cM/Mb
0 cM/Mb
4
2
0
$-\log_{10}P$
HNMT +
LOC101928273 -
R2 with most significant discovery phase SNP
1.00
0.75
0.50
0.25
137500000
137750000
138000000
138250000
138500000
Position in BP on Chromosome 2 (hg19)

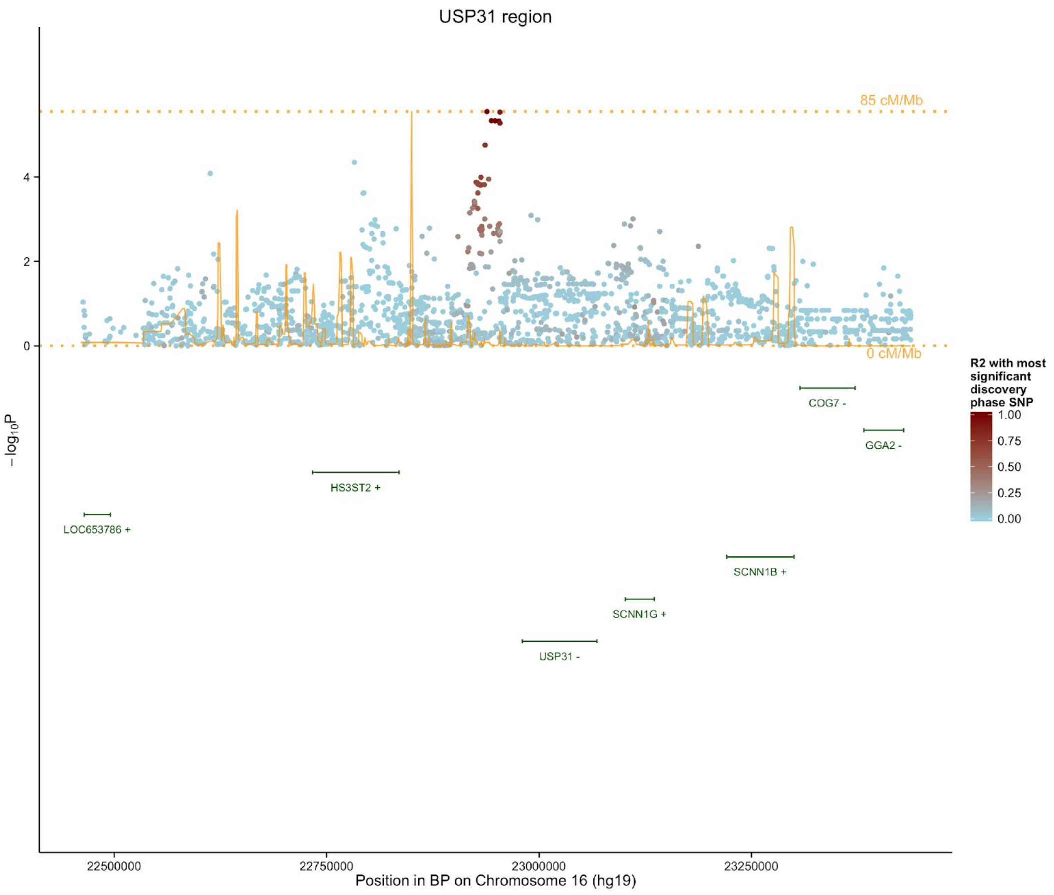

USP31 region
85 cM/Mb
0 cM/Mb
4
2
0
−log10P
R2 with most significant discovery phase SNP
1.00
0.75
0.50
0.25
0.00
COG7 -
GGA2 -
HS3ST2 +
LOC653786 +
SCNN1B +
SCNN1G +
USP31 -
22500000
22750000
23000000
23250000
Position in BP on Chromosome 16 (hg19)

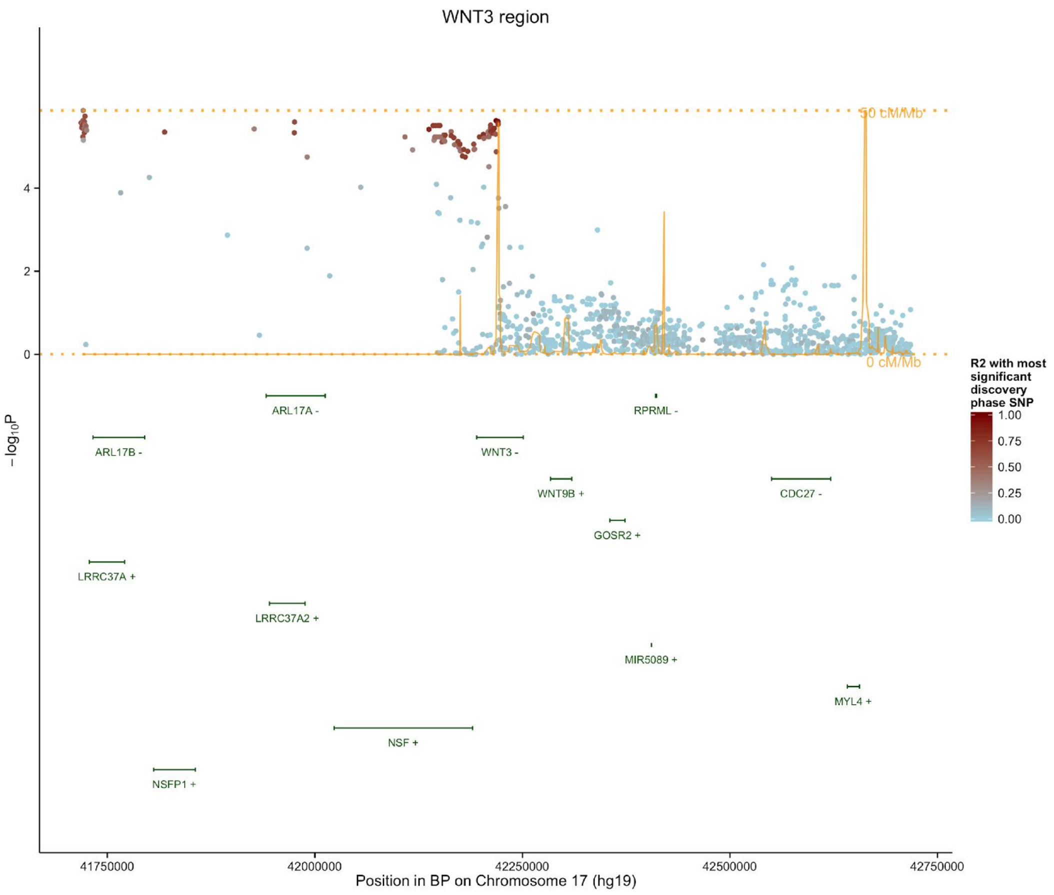

WNT3 region
50 cM/Mb
0 cM/Mb
4
2
0
−log10P
ARL17A -
RPRML -
ARL17B -
WNT3 -
WNT9B +
CDC27 -
GOSR2 +
LRRC37A +
LRRC37A2 +
MIR5089 +
MYL4 +
NSF +
NSFP1 +
41750000
42000000
42250000
42500000
42750000
Position in BP on Chromosome 17 (hg19)
R2 with most significant discovery phase SNP
1.00
0.75
0.50
0.25
0.00

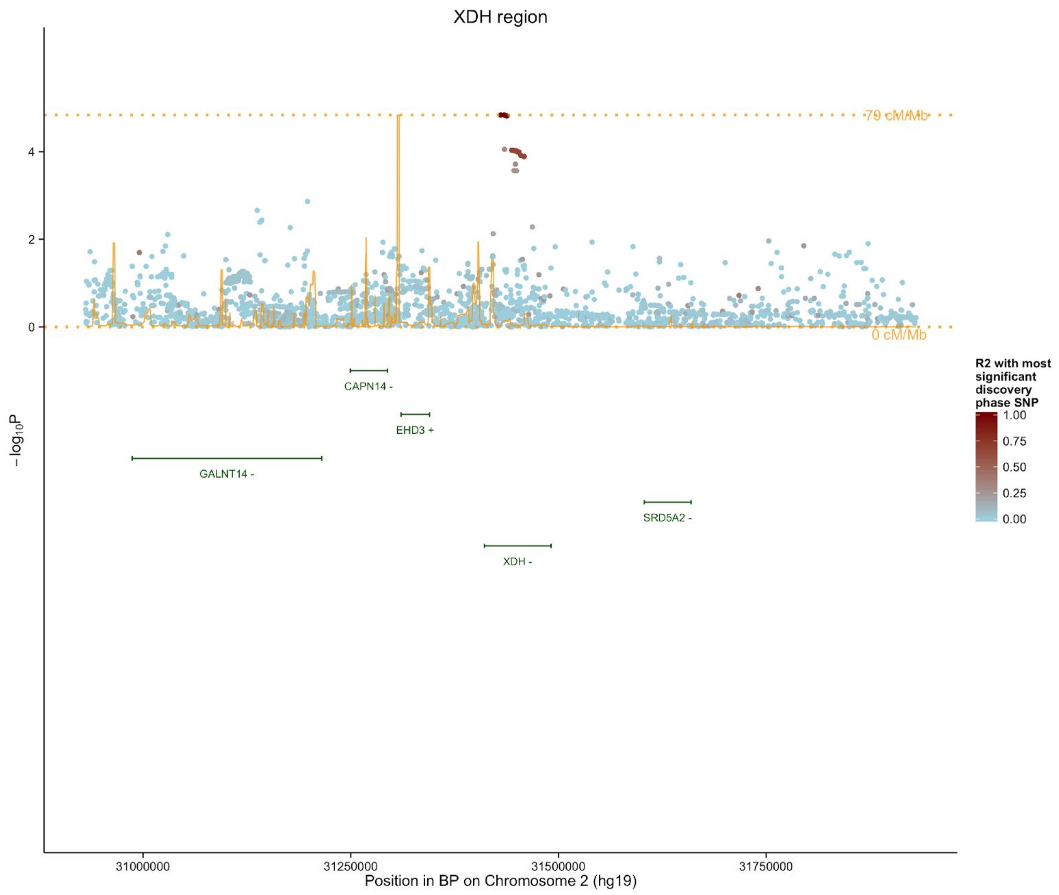

XDH region
79 cM/Mb
0 cM/Mb
4
2
0
−log10P
CAPN14 -
EHD3 +
GALNT14 -
SRD5A2 -
XDH -
31000000
31250000
31500000
31750000
Position in BP on Chromosome 2 (hg19)
R2 with most significant discovery phase SNP
1.00
0.75
0.50
0.25
0.00
