# Supplementary material for: A genome-wide association study in multiple system atrophy
Source: Neurology. 2016 Oct 11;87(15):1591–8. doi: 10.1212/WNL.0000000000003221 (PMC5067544; doi:10.1212/WNL.0000000000003221)
Supplement: Coinvestigators [file supp_WNL.0000000000003221_Pellecchia_Coinvestigators.docx]

**CO-INVESTIGATORS**

European multiple system atrophy study group (EMSA) co-investigators (in alphabetical order):

Angelo Antonini, MD, PhD (Parkinson and Movement Disorders Unit, IRCCS Hospital San Camillo, Venice, Italy; patient recruitment); Yakov Balash, MD, PhD (Movement Disorders Unit and Neuroautonomic service, Department of Neurology, Tel-Aviv Sourasky Medical Center, Tel-Aviv, Israel; patient recruitment); Paolo Barone, MD, PhD (Università degli Studi di Salerno, Dipartimento di Medicina e Chirurgia, Via Giovanni Paolo II, Salerno, Italy; patient recruitment); Daniela Berg, MD (Department of Neurodegenerative Diseases, Hertie Institute for Clinical Brain Research, Tübingen, Germany and German Center for Neurodegenerative diseases (DZNE), Tübingen, Germany; patient recruitment); Roberta Biundo, PhD (Parkinson and Movement Disorders Unit, IRCCS Hospital San Camillo, Venice, Italy; patient recruitment); Sylvia Bösch, MD (Institute of Neurology, Medical University Innsbruck, Austria; patient recruitment); David Brooks, DSc (Department of Neurology, Aarhus University Hospital, Aarhus, Denmark; patient recruitment); Hana Brožová, MD (Department of Neurology and Centre of Clinical Neuroscience, Charles University in Prague, First Faculty of Medicine and General University Hospital in Prague, Czech Republic; patient recruitment); Giovanna Calandra-Buonaura, MD, PhD (IRCCS Institute of Neurological Sciences of Bologna and University of Bologna, Bologna, Italy; patient recruitment); Margherita Canesi, MD (Parkinson Institute, Istituti Clinici di Perfezionamento, Milan, Italy); Sabina Capellari, MD (IRCCS Institute of Neurological Sciences of Bologna and University of Bologna, Bologna, Italy; patient recruitment); Carlo Colosimo, MD (Department of Neurology and Psychiatry, Sapienza University, Rome, Italy; patient recruitment); Pietro Cortelli, MD (IRCCS Institute of Neurological Sciences of Bologna and University of Bologna, Bologna, Italy; patient recruitment); Erik Danielsen, MD, PhD (Department of Neurology, Aarhus University Hospital, Aarhus, Denmark; patient recruitment); Ruth Djaldetti, MD (Department of Neurology, Rabin Medical Center, Beilinson Campus, Petach Tikva, Israel; patient recruitment); Giovanni Fabbrini, MD (Department of Neurology and Psychiatry, University Sapienza, Rome, Italy; IRCSS Neuromed Institute, Pozzilli, Italy; patient recruitment); Alessandra Fanciulli, MD (Institute of Neurology, Medical University Innsbruck, Austria; patient recruitment); Alexandra Foubert-Samier, MD (Centre de référence atrophie multisystématisée, CHU de Bordeaux, Pessac, France; patient recruitment); Alexander Gerhard, MD (Institute of Brain, Behaviour and Mental Health, The University of Manchester, Manchester, United Kingdom; patient recruitment); Nir Giladi, MD (Movement Disorders Unit and Neuroautonomic service, Department of Neurology, Tel-Aviv Sourasky Medical Center, Tel-Aviv, Israel; patient recruitment); Tanya Gurevich, MD (Movement Disorders Unit and Neuroautonomic service, Department of Neurology, Tel-Aviv Sourasky Medical Center, Tel-Aviv, Israel; patient recruitment); Oskar Hansson, MD, PhD (Clinical Memory Research Unit, Department of Clinical Sciences, Lund University, Lund, Sweden; Department of Neurology, Johns Hopkins Hospital, Baltimore, MD, USA; patient recruitment); Jiri Klempir, MD, PhD (Department of Neurology and Centre of Clinical Neuroscience, Charles University in Prague, First Faculty of Medicine and General University Hospital in Prague, Czech Republic; patient recruitment); Thomas Klockgether, MD, PhD (Department of Neurology, University Hospital of Bonn Medical Center, Bonn, Germany; German Center for Neurodegenerative Diseases, University Hospital of Bonn Medical Center, Bonn, Germany; patient recruitment); Magda Kobierowska, MD (Department of Neurology, St. Adalbert Hospital, Gdańsk, Poland and Department of Neurological and Psychiatric Nursing, Medical University of Gdańsk, Gdańsk, Poland; patient recruitment); Vladimir Kostic, MD (Institute of Neurology Clinical Centre of Serbia, School of Medicine, University of Belgrade, Belgrade, Serbia; patient recruitment); Florian Krismer, MD (Institute of Neurology, Medical University Innsbruck, Austria; patient recruitment); Milica Ječmenica Lukić, MD (Institute of Neurology Clinical Centre of Serbia, School of Medicine, University of Belgrade, Belgrade, Serbia; patient recruitment); Philipp Mahlknecht, MD (Institute of Neurology, Medical University Innsbruck, Austria; patient recruitment); Yael Manor, MA (Movement Disorders Unit and Neuroautonomic service, Department of Neurology, Tel-Aviv Sourasky Medical Center, Tel-Aviv, Israel; patient recruitment); Andrea Marcante, MD (Parkinson and Movement Disorders Unit, IRCCS Hospital San Camillo, Venice, Italy; patient recruitment); Maria Jose Marti, MD (Hospital Clinic, University of Barcelona, Barcelona, Spain; patient recruitment); Giuseppe Meco, MD (Department of Neurology and Psychiatry and Research Centre of Social Diseases (CIMS), Sapienza University, Rome, Italy; patient recruitment); Christer Nilsson, PhD (Clinical Memory Research Unit, Department of Clinical Sciences, Lund University, Lund, Sweden; Department of Neurological Sciences, University Federico II, Napoli, Italy; patient recruitment); Karen Østergaard, MD (Department of Neurology, Aarhus University Hospital, Aarhus, Denmark; patient recruitment); Nicola Pavese, MD, PhD (Department of Neurology, Aarhus University Hospital, Aarhus, Denmark; patient recruitment); Anne Pavy-LeTraon, MD, PhD (Clinical Investigation Center CIC1436, Departments of Clinical Pharmacology and Neurosciences, INSERM and University Hospital of Toulouse, Faculty of Medicine, Toulouse, France; patient recruitment); Mariya Ivanova Petrova, MD (Department of Neurology, University Hospital (UH) Alexandrovska, Sofia, Bulgaria; patient recruitment); Carlo Purcaro, MD (Department of Neurology and Psychiatry and Research Centre of Social Diseases (CIMS), Sapienza University, Rome, Italy; patient recruitment); Heinz Reichmann, MD (Department of Neurology, Dresden University of Technology, Fetscherstraße 74, Dresden, Germany; patient recruitment); Vincent Ries, MD (Centre of Nervous Diseases, Philipps-University of Marburg, Marburg, Germany; patient recruitment); Olaf Riess, MD (Department of Neurodegenerative Diseases, Hertie Institute for Clinical Brain Research, Tübingen, Germany and German Center for Neurodegenerative diseases (DZNE), Tübingen, Germany; patient recruitment); Nahum Roisen, MD (Department of Neurology, Rabin Medical Center, Beilinson Campus, Petach Tikva, Israel; patient recruitment); Alfonso Rubino, MD (Department of Neurology and Psychiatry and Research Centre of Social Diseases (CIMS), Sapienza University, Rome, Italy; patient recruitment); Evžen Růžička, MD, DSc (Department of Neurology and Centre of Clinical Neuroscience, Charles University in Prague, First Faculty of Medicine and General University Hospital in Prague, Czech Republic; patient recruitment); Luisa Sambati, MD, PhD (IRCCS Institute of Neurological Sciences of Bologna, University of Bologna, Bologna, Italy; patient recruitment); Christoph Scherfler, MD (Institute of Neurology, Medical University Innsbruck, Austria; patient recruitment); Michal Schinwelski, MD (Department of Neurology, St. Adalbert Hospital, Gdańsk, Poland and Department of Neurological and Psychiatric Nursing, Medical University of Gdańsk, Gdańsk, Poland; patient recruitment); Susanne A. Schneider, MD (Department of Neurodegenerative Diseases, Hertie Institute for Clinical Brain Research, Tübingen, Germany and German Center for Neurodegenerative diseases (DZNE), Tübingen, Germany; Department of Neurology, University of Kiel, Kiel, Germany; patient recruitment); Anette Schrag, MD (UCL Institute of Neurology, University College London, London, United Kingdom; patient recruitment); Klaus Seppi, MD (Institute of Neurology, Medical University Innsbruck, Austria; patient recruitment); Herzl Shabtai, MD (Movement Disorders Unit and Neuroautonomic service, Department of Neurology, Tel-Aviv Sourasky Medical Center, Tel-Aviv, Israel; patient recruitment); Manu Sharma, PhD (Department of Neurodegenerative Diseases, Hertie Institute for Clinical Brain Research, Tübingen, Germany and German Center for Neurodegenerative diseases (DZNE), Tübingen, Germany; patient recruitment); María Sierra, MD (Service of Neurology, University Hospital Marqués de Valdecilla (IDIVAL), University of Cantabria (UC), Santander, Spain; patient recruitment); Emilia Sitek, MA, PhD (Department of Neurology, St. Adalbert Hospital, Gdańsk, Poland and Department of Neurological and Psychiatric Nursing, Medical University of Gdańsk, Gdańsk, Poland; patient recruitment); Jarosław Sławek, MD (Department of Neurology, St. Adalbert Hospital, Gdańsk, Poland and Department of Neurological and Psychiatric Nursing, Medical University of Gdańsk, Gdańsk, Poland; patient recruitment); Francesca Del Sorbo, MD (Istituto Clinico Humanitas, Università Cattolica del Sacro Cuore, Milano, Italy; patient recruitment); Iva Stankovic, MD (Institute of Neurology Clinical Centre of Serbia, School of Medicine, University of Belgrade, Belgrade, Serbia; patient recruitment); Flavio Di Stasio, MD (Department of Neurology and Psychiatry, Sapienza University, Rome, Italy; patient recruitment); Francois Tison, MD, PhD (Centre de référence atrophie multisystématisée, CHU de Bordeaux, Pessac, France; patient recruitment); Latchezar Dintchev Traykov, MD, PhD, DSc (Department of Neurology, University Hospital (UH) Alexandrovska, Sofia, Bulgaria; patient recruitment); Bart P. van de Warrenburg, MD, PhD (Radboud University Nijmegen Medical Centre, Nijmegen, Netherlands; patient recruitment)
