# Supplementary material for: A genome-wide association study in multiple system atrophy
Source: Neurology. 2016 Oct 11;87(15):1591–8. doi: 10.1212/WNL.0000000000003221 (PMC5067544; doi:10.1212/WNL.0000000000003221)
Supplement: Accompanying Editorial [file supp_WNL.0000000000003221_1530.pdf]

Genetics of multiple system atrophy

Back to square one?

Oliver Bandmann, MD, PhD, FAAN
Adriano Chio, MD, FAAN

Correspondence to Dr. Bandmann: o.bandmann@sheffield.ac.uk

Neurology® 2016;87:1530–1531

Multiple system atrophy (MSA) typically presents with a combination of parkinsonism, cerebellar ataxia, and autonomic failure.[1] Its incidence is estimated at 3 cases per 100,000 patients per year for people aged 50–99 years.[2] Median survival from symptom onset is less than 10 years, while time from diagnosis to death is often considerably shorter. Both Parkinson disease (PD) and MSA are α-synucleinopathies but differ neuropathologically, in that α-synuclein aggregates predominantly in oligodendroglial cells in MSA rather than in neurons, as observed in PD. Similar to PD, there is also evidence of a prion-like spread of pathologic α-synuclein in MSA brains, with some studies suggesting α-synuclein strain specificity for the different neurodegenerative disorders.[3,4] These and many other studies have provided important new insight into the mechanisms underlying the observed neurodegeneration but, crucially, have not revealed much about the etiology of MSA.

The discovery of monogenically inherited genes such as *LRRK2* or *parkin* has revolutionized PD research but similar discoveries of Mendelian MSA genes have been noticeably absent. Some neurogenetic disorders such as spinocerebellar ataxia type 1 or fragile X–associated tremor ataxia syndrome may occasionally phenocopy MSA, but this is rare and unlikely to be relevant for our understanding of MSA etiology. Mutations in *COQ2*, essential for the biosynthesis of coenzyme Q10, were detected in 2 Japanese multiplex families with MSA; in addition, an association of sporadic MSA with naturally occurring *COQ2* variants was reported, but these results were not confirmed in other populations and appear to be private mutations.[1] The most plausible genetic finding in the synucleinopathy MSA to date was an association with 2 single nucleotide polymorphisms (SNP) at the α-synuclein (*SNCA*) locus.[5] However, all genetic association studies in MSA carried out to date were undertaken in comparatively small patient and control cohorts with a limited number of genetic markers only.

In this issue of *Neurology*®, Sailer et al.[6] report their findings in a considerably larger genome-wide association study (GWAS) in MSA. More than 340,000 SNPs were analyzed for possible association with MSA in DNA samples from 1,030 MSA cases and compared to previously published genotype data from 3,864 controls. Crucially, the previously reported associations with *SNCA* and *COQ2* were refuted. No other MSA risk loci were identified that remained significant after stringent multiple testing correction.

However, a number of potentially interesting regions emerged at $p < 1 \times 10^{-6}$, including the genes *FBXO47*, *ELOL7*, *EDN1*, and *MAPT*. What do we know about these genes and how do they fit with our current knowledge about MSA? *FBXO47* belongs to a large family of genes with an F-box motif; several of the genes belonging to this family are involved in ubiquitin-dependent protein degradation.[7] Impaired protein degradation may contribute to glial α-synuclein aggregation as observed in MSA. *ELOLV7* elongates very long chain fatty acids.[8] Lipid dysfunction has been implicated in the pathogenesis of MSA via impaired myelin synthesis and maintenance by oligodendrocytes. *EDN1* belongs to the endothelin gene family that regulates the maintenance of vascular tone. This provides a potential link with autonomic function in MSA. Among the most highly associated regions is the *MAPT* locus, despite MSA not being a classical tauopathy. While these findings are of interest, they need to be seen as hypothesis-generating rather than definitive: The associated SNPs are not in coding parts of the respective genes and therefore of uncertain functional relevance. Indeed, they may only exert an effect via transcriptional regulation of an adjacent or more distant gene. The putative links for any of these genes with known pathomechanisms leading to MSA are at best tentative.

To put the study by Sailer et al. into context, it might help to compare it to GWAS in PD, where a recent meta-analysis relied on a common set of nearly 8 million genetic variants across 13,708 cases and 95,282 controls to identify or confirm 24 risk loci for PD.[9] The estimated heritability of MSA is likely even lower than in PD.[10] Accordingly, considerably larger MSA cohorts than the study by Sailer et al. may be required to firmly establish or refute the

See page 1591

From Sheffield Institute for Translational Neuroscience (SITraN) (O.B.), University of Sheffield, UK; and Rita Levi Montalcini Department of Neuroscience (A.C.), University of Torino, Turin, Italy.

Go to Neurology.org for full disclosures. Funding information and disclosures deemed relevant by the authors, if any, are provided at the end of the editorial.

© 2016 American Academy of Neurology
© 2016 American Academy of Neurology. Unauthorized reproduction of this article is prohibited.

putative association of MSA with *FBXO47*, *ELOL7*, *EDN1*, and *MAPT*, and to identify novel risk loci if the same genetic techniques were applied. However, it will be challenging to recruit such large cohorts for the much rarer orphan disease MSA. Interestingly, the authors of this study were able to collect 699 patients with MSA of European ancestry owing to the collaboration of more than 30 American and European movement disorders clinics. Other approaches, such as whole exome or genome sequencing, may be worth exploring, but have their own inherent limitations. More rapid functional assessment of putative risk variants, including the use of novel in vitro and in vivo models, will help to assess the likely relevance of putative risk variants more quickly and establish their biological relevance (rather than just relying on statistical significance).

Critics of GWAS may argue that the observed increased risk is typically very small even for firmly established risk loci in PD or other neurodegenerative disorders such as Alzheimer disease, and therefore unlikely to translate into relevant insight or even novel therapeutic approaches. However, GWAS can help to identify novel functional networks that may be amenable to therapeutic intervention at the hub level. Ultimately, in the absence of any real progress in other areas investigating the etiology of MSA, genetic studies including future, larger GWAS are currently our best bet to obtain a better understanding of the causes of MSA. This will hopefully help towards the eventual discovery of neuroprotective treatment for this currently relentlessly progressive condition.

STUDY FUNDING

No targeted funding reported.

DISCLOSURE

O.B. receives grants from Parkinson's UK and the Michael J. Fox Foundation and is a member of the Editorial Board of *Neurology*. A.C. is a member of the Editorial Board of *Amyotrophic Lateral Sclerosis and Frontotemporal Degeneration*. Go to Neurology.org for full disclosures.

REFERENCES

1. Fanciulli A, Wenning GK. Multiple-system atrophy. N Engl J Med 2015;372:249–263.
2. Bower JH, Maraganore DM, McDonell SK, Rocca WA. Incidence of progressive supranuclear palsy and multiple system atrophy in Olmsted County, Minnesota, 1976 to 1990. Neurology 1997;49:1284–1288.
3. Prusiner SB, Woerman AL, Mordes DA, et al. Evidence for alpha-synuclein prions causing multiple system atrophy in humans with parkinsonism. Proc Natl Acad Sci USA 2015;112:E5308–E5317.
4. Peelaerts W, Bousset L, Van der Perren A, et al. Alpha-synuclein strains cause distinct synucleinopathies after local and systemic administration. Nature 2015;522:340–344.
5. Scholz SW, Houlden H, Schulte C, et al. *SNCA* variants are associated with increased risk for multiple system atrophy. Ann Neurol 2009;65:610–614.
6. Sailer A, Scholz SW, Nalls MA, et al. A genome-wide association study in multiple system atrophy. Neurology 2016;87:1591–1598.
7. Simon-Kayser B, Scoul C, Renaudin K, et al. Molecular cloning and characterization of FBXO47, a novel gene containing an F-box domain, located in the 17q12 band deleted in papillary renal cell carcinoma. Genes Chromosomes Cancer 2005;43:83–94.
8. Naganuma T, Sato Y, Sassa T, Ohno Y, Kihara A. Biochemical characterization of the very long-chain fatty acid elongase ELOVL7. FEBS Lett 2011;585:3337–3341.
9. Nalls MA, Pankratz N, Lill CM, et al. Large-scale meta-analysis of genome-wide association data identifies six new risk loci for Parkinson's disease. Nat Genet 2014;46:989–993.
10. Federoff M, Price TR, Sailer S, et al. Genome-wide estimate of the heritability of multiple system atrophy. Parkinsonism Relat Disord 2016;22:35–41.

© 2016 American Academy of Neurology. Unauthorized reproduction of this article is prohibited.
